# Automated deep learning-based assessment of tumour-infiltrating lymphocyte density determines prognosis in colorectal cancer

**DOI:** 10.1186/s12967-025-06254-3

**Published:** 2025-03-10

**Authors:** Joshua Millward, Zhen He, Aiden Nibali, Dmitri Mouradov, Lisa A Mielke, Kelly Tran, Angela Chou, Nicholas J Hawkins, Robyn L Ward, Anthony J Gill, Oliver M Sieber, David S Williams

**Affiliations:** 1 School of Computing, Engineering and Mathematical Sciences, La Trobe University, Melbourne, Australia; 2 Personalised Oncology Division, The Walter and Eliza Hall Institute of Medical Research, Melbourne, Australia; 3 Department of Medical Biology, The University of Melbourne, Melbourne, Australia; 4 Olivia Newton-John Cancer Research Institute, Melbourne, Australia; 5 La Trobe University School of Cancer Medicine, Melbourne, Australia; 6 Department of Anatomical Pathology, NSW Health Pathology, Royal North Shore Hospital, Sydney, Australia; 7 Sydney Medical School, University of Sydney, Sydney, Australia; 8 Cancer Diagnosis and Pathology Group, Kolling Institute of Medical Research, Royal North Shore Hospital, Sydney, Australia; 9 School of Biomedical Sciences, UNSW Sydney, Sydney, Australia; 10 Faculty of Medicine and Health, University of Sydney, Sydney, Australia; 11 Department of Surgery, The University of Melbourne, Melbourne, Australia; 12 Department of Biochemistry and Molecular Biology, Monash University, Melbourne, Australia; 13 Department of Anatomical Pathology, Austin Health, Melbourne, Australia

**Keywords:** Computational pathology, Image analysis, Tissue segmentation, Cell detection

## Abstract

**Background:**

The presence of tumour-infiltrating lymphocytes (TILs) is a well-established prognostic biomarker across multiple cancer types, with higher TIL counts being associated with lower recurrence rates and improved patient survival. We aimed to examine whether an automated intraepithelial TIL (iTIL) assessment could stratify patients by risk, with the ability to generalise across independent patient cohorts, using routine H&E slides of colorectal cancer (CRC). To our knowledge, no other existing fully automated iTIL system has demonstrated this capability.

**Methods:**

An automated method employing deep neural networks was developed to enumerate iTILs in H&E slides of CRC. The method was applied to a Stage III discovery cohort (*n* = 353) to identify an optimal threshold of 17 iTILs per-mm^2^ tumour for stratifying relapse-free survival. Using this threshold, patients from two independent Stage II-III validation cohorts (*n* = 1070, *n* = 885) were classified as “TIL-High” or “TIL-Low”.

**Results:**

Significant stratification was observed in terms of overall survival for a combined validation cohort univariate (HR 1.67, 95%CI 1.39–2.00; *p* < 0.001) and multivariate (HR 1.37, 95%CI 1.13–1.66; *p* = 0.001) analysis. Our iTIL classifier was an independent prognostic factor within proficient DNA mismatch repair (pMMR) Stage II CRC cases with clinical high-risk features. Of these, those classified as TIL-High had outcomes similar to pMMR clinical low risk cases, and those classified TIL-Low had significantly poorer outcomes (univariate HR 2.38, 95%CI 1.57–3.61; *p* < 0.001, multivariate HR 2.17, 95%CI 1.42–3.33; *p* < 0.001).

**Conclusions:**

Our deep learning method is the first fully automated system to stratify patient outcome by analysing TILs in H&E slides of CRC, that has shown generalisation capabilities across multiple independent cohorts.

**Supplementary Information:**

The online version contains supplementary material available at 10.1186/s12967-025-06254-3.

## Background

Colorectal cancer (CRC) was the third most commonly diagnosed cancer and the second leading cause of cancer-related death in 2020 [1]. The most commonly used staging system for classifying cancer patients into prognostic groups is the American Joint Committee on Cancer’s TNM (tumour, node, metastasis) classification, which is used to guide treatment approach [2]. Whilst a well-established system, studies have demonstrated that other biomarkers can serve as prognostic indicators, and in some cases, outperform the TNM system in predicting prognosis.

One such biomarker is the presence of tumour-infiltrating lymphocytes (TILs). For multiple cancer types, higher TIL counts are associated with reduced recurrence rates and enhanced patient survival [3–8]. In the case of CRC, a strong correlation exists between the presence of TILs and either DNA mismatch repair deficiency (MMR) or polymerase epsilon mutation, molecular subtypes associated with high tumour mutational burden, and response to checkpoint inhibitor therapy [9–11].

One approach for the standardised assessment of TILs in CRC is by the Immunoscore [12, 13], which was found to be a strong predictor of survival, involving quantifying CD3 + and CD8 + T lymphocytes in the tumour centre and invasive margin using immunohistochemistry (IHC) stained samples. Since Immunoscore requires samples to be submitted to a specialised lab for processing, factors of cost and test turnaround time limit access to this assay in routine clinical practice.

H&E staining is standard in routine pathology, and it is common to see works that attempt to manually score TILs from these samples [6–8, 14, 15]. Differing methods have been applied, and there is currently no recommended method for scoring TILs in the International Collaboration on Cancer Reporting guidelines for CRC [16]. Manual TIL assessments are time consuming, require pathologist training, and are subject to inter- and intra-rater variability due to difficulties in identifying TILs from H&E alone [17]. These limitations have been well studied in breast cancer [18–20], and present an opportunity for objective, repeatable computer-based automated scoring methods. Recent works predominantly utilise deep learning algorithms for automated TIL scoring [21–39].

To the best of our knowledge, there are few existing works that automate the assessment of TILs in H&E-stained CRC whole slide images (WSIs) [27–31]. These works were successful in estimating TIL densities and finding relationships to patient survival, however two main limitations are present amongst these studies.

It is well established that deep learning algorithms often fail to generalise to new data with different appearance features than those the algorithm was trained on [40]. In the context of computational pathology, where algorithms are expected to work across multiple centres, different appearance features can arise due to challenges relating to the standardisation within pathology across centres. These variations can be due to factors such as differences in staining protocols or scanners [41]. This is why it is crucial that algorithms are tested in multiple patient cohorts from different centres to demonstrate they can generalise to new patient cohorts without the need for further tuning. Of the existing work, only one [28] has evaluated their algorithm in separate independent cohorts without requiring further tuning. This raises the question of how well the other studies [29–31] would generalise.

Despite evaluating their algorithm on patients (*n* = 938) from other cohorts, the work by Pai et al. [28] has other limitations in that it is not fully automated, requiring the tumour bed to first be manually identified by a pathologist. Additionally, it requires the use of commercial software, which may limit the accessibility of their algorithm.

The second limitation among the existing work is that some approaches make classifications on patches of tissue to localise tumour [29, 30] or TILs [29], limiting the accuracy of intraepithelial TIL (iTIL) density quantification (Supplementary Figure S1). Patch-based classification for tumour localisation provides a coarser view of the tumour. For example, a patch consisting of 51% tumour and another consisting of 95% tumour could both be classified as tumour patches, thus limiting the accuracy of any iTIL scoring method that needs to know if a TIL resides in tumour or stroma. Similarly, performing TIL scoring at the patch grain would result in classifying an entire patch as TIL positive when only a single TIL is present, thus precluding accurate estimations of TIL densities [41].

To address the limitations of existing work in TIL scoring for H&E-stained CRC WSIs, we have developed a fully automated deep learning method that performs both tumour tissue identification and TIL detection at pixel-level granularity. Our method computes a score that quantifies the density of iTILs per-mm^2^ tumour. After tuning the iTIL score cut-off threshold on a single cohort from Austin Health (Austin-CRC, *n* = 353), we found the same threshold was able to stratify patients into high/low risk groups across two large independent cohorts, from the Royal North Shore Hospital (RNSH-CRC, *n* = 1070), and the Molecular and Cellular Oncology study (MCO-CRC, *n* = 885). This result indicates that our method can be used on WSIs from a new hospital or cohort without the need for further model training or the use of additional survival data to tune the cut-off.

## Methods

### Datasets

Our TIL scoring method is built from three deep neural network models. These models were trained and evaluated using annotated H&E-stained WSIs from 59 primary CRC tumours, consisting of both Stage I and Stage IV cases, diagnosed at Austin Health, in addition to 47 WSIs from the PAIP2020^*^^1^ Grand Challenge, 46 privately annotated WSIs from the public CPTAC-COAD^†^^2^[42, 43] cohort, and all data from the Lizard [44] dataset. WSIs used for model training were not used for survival analysis.

Three independent cohorts of H&E-stained CRC WSIs were used for survival analysis to evaluate the prognostic power of our TIL scoring method: Austin-CRC (*n* = 353, 516 WSIs), RNSH-CRC (*n* = 1070), and MCO-CRC (*n* = 885). All cases were scanned at 40× magnification. The Austin-CRC cohort consisted of Stage III cases diagnosed between 1997 and 2017 at Austin Health, scanned on a Leica Biosystems Aperio AT2 scanner. 93 patients in this cohort had at least 2 WSIs available. The median follow-up time was 33.1 months in terms of relapse-free survival (RFS) (52.8 months for censored patients). The RNSH-CRC cohort consisted of Stage II and III cases diagnosed between 1998 and 2018 at the Royal North Shore Hospital, scanned using a Hamamatsu NanoZoomer S210 Scanner. The median follow-up time was 37.6 months in terms of overall survival (OS) (RFS data was not collected), and 45.5 months for censored patients. The MCO-CRC^‡^^3^ cohort was provided by the Molecular and Cellular Oncology research group [45–47] and consisted of Stage II and III cases scanned on a Leica Biosystems Aperio XT scanner. The median follow-up time was 48.2 months for RFS (60 months for censored patients), and 50 months for OS (60 months for censored patients). Supplementary Table S1 describes the clinicopathological features of each cohort. Supplementary Figure S2 illustrates the breakdown of datasets used for model training and survival analysis.

This study was approved by human research ethics committees for each site, with a waiver of consent for the Austin and RNSH cohorts (HREC/80030/Austin-2021).

### Method overview

**Fig. 1:**
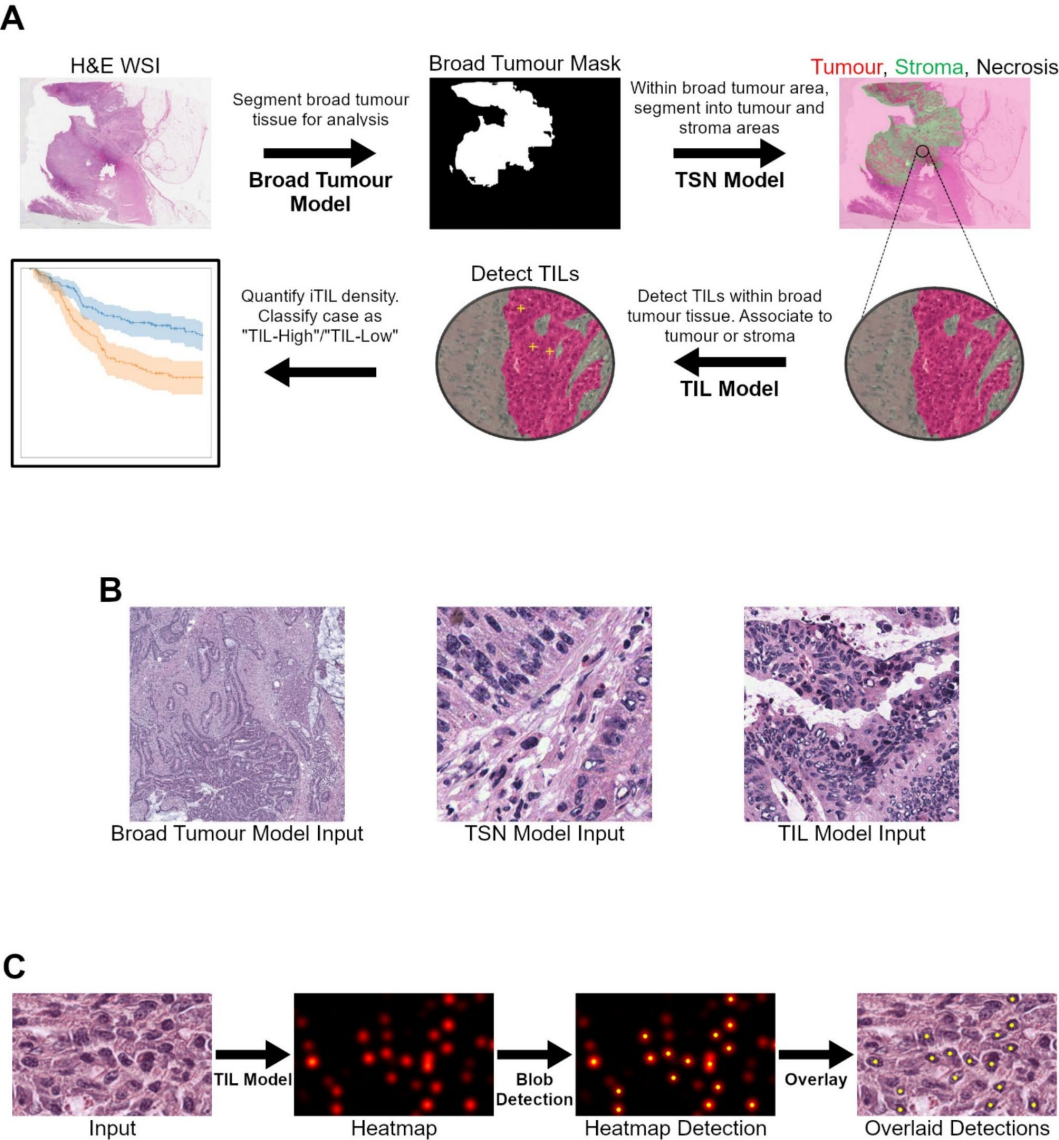
(**A**) Given a whole slide image, our proposed AI iTIL scoring method first performs coarse semantic segmentation to identify broadly cancerous tissue. Within the cancerous region, one fine-grained model segments areas of tumour, stroma, and necrosis and another detects TILs. A summary iTIL score is calculated from the median quantification of TIL density associated with tumour regions, then thresholded to stratify patient outcome. (**B**) 512 × 512px regions fed to each model with different amounts of context, representing tissue areas of 2.048mm^2^ at 4MPP, 256 µm^2^ at 0.5 MPP, and 128 µm^2^ at 0.25 MPP respectively. (**C**) Given an input image, our TIL model generates a heatmap consisting of circular, blob-like areas which correspond to the likelihood that each pixel belongs to a TIL. Using blob detection, we extract a set of point coordinates (shown with yellow dots) corresponding to TIL locations, which rejects the detection of TILs in areas of low likelihood (seen as faint blobs in the heatmap)

Figure 1 A outlines our automated iTIL scoring method. Given a H&E-stained CRC WSI, we (1) separate broadly cancerous tissue from normal tissue and background via semantic segmentation, (2) separate tumour, stroma, and necrosis within cancerous tissue via semantic segmentation, and (3) detect TILs within cancerous tissue via point detection. TILs associated with tumour (iTILs) are then extracted and quantified to derive a summary ‘AI iTIL score’ based on the number of iTILs per-mm^2^ tumour. Each stage is described in the following sections, with additional details on hyperparameters, data augmentation, and performing inference on a WSI provided in the supplementary material. All models were trained using the open-source PyTorch [48] package.

### Broad tumour segmentation

To enable a fully automated iTIL quantification that ensures TIL density estimates are only performed in areas where cancer is present, we first perform broad tumour segmentation to separate the background and normal tissue from cancerous tissue. Existing works either use manual pathologist annotations for this task [28, 37, 38] or classify patches of tissue across the entire WSI and extract the ‘tumour’ patches for further analysis [29, 30]. In contrast, we use a semantic segmentation model to automatically label each pixel of the WSI as belonging to the tumour or not.

We trained a SegFormer-B0 model [49] using data from two different sources – 47 WSIs from the PAIP2020 Grand Challenge, and 51 WSIs designated as model training data from the Austin-CRC cohort, consisting of Stage I and IV cases. Annotations were made in the form of polygons surrounding cancer tissue. In the Austin-CRC cohort, these were made by pathologist DW using the QuPath software package [50], whilst WSIs from the PAIP2020 dataset have publicly available annotations.

Our main motivation for selecting SegFormer lies in its transformer-based architecture, which incorporates a multiscale attention mechanism to extract both fine and coarse-grained features. This allows more effective utilisation of the contextual information within the images compared to traditional convolution-based architectures. For tissue segmentation, this allows the model to focus on detailed information about the tissue structures, such as their general shape, while also observing coarse-level features, such as how these structures are organised and interact with each other. Additionally, previous studies have demonstrated SegFormer’s better performance [51, 52], further justifying its use. Several variants of SegFormer are available, from B0 to B5, with higher numbers indicating greater model complexity and parameter counts. For this task, we chose the B0 variant, as we found the other variants were more computationally expensive without improving segmentation results.

Images were scaled to a resolution of 4 microns per pixel (MPP) (2.5× magnification), and Macenko stain normalisation [53] was applied to all image data to lessen the impact of inter-scanner variability. At training time, random crops of 512 × 512px were fed to the model, providing 2.048mm^2^ of context (Fig. 1B). Each crop had a suite of augmentations randomly applied, including rotation, scaling, flipping, Gaussian blurring, colour jittering, and downscaling. Cross Entropy loss was used between predicted and ground truth segmentation masks to train the model. At inference time, the WSI was broken up into 512 × 512px tiles, then per-tile predictions were reconstructed to generate a single prediction for the entire WSI.

### Tumour/stroma/necrosis segmentation

Within detected cancerous tissue, we segment regions into tumour, stroma, and necrosis (TSN) to enable associations of TILs to tumour (iTILs). We trained a SegFormer-B0 model on 643 varying sized annotated regions from 105 WSIs. 46 WSIs were from the CPTAC-COAD cohort, whilst 59 were from the Austin-CRC training cohort. All data was manually annotated by pathologist DW in the QuPath software package.

Image data was scaled to 20× magnification (Fig. 1B), giving the model a more fine-grained view than the broad tumour model. With the exception of the learning rate and number of epochs, the training procedure for TSN segmentation was the same as for broad tumour segmentation. At inference time, TSN segmentation was only applied within areas of broadly cancerous tissue (as determined by the broad tumour segmentation model).

### TIL detection

We used a SegFormer-B1 model adapted for point-based detection to identify TILs within cancerous tissue. We selected the B1 variant of SegFormer as it provided the best trade-off between computation time and TIL detection performance, outperforming the B0 variant in TIL detection metrics, while achieving results similar to the more complex variants. We first pre-trained the model on a large public dataset with nuclei annotations [44], then finetuned the model on the Austin-CRC and CPTAC-COAD training datasets. Annotations on the Austin-CRC and CPTAC-COAD datasets were made by pathologist DW in the form of point annotations on each TIL in a given annotated region (tumour or stroma). In total, 20,599 TILs were annotated by DW across 368 regions from 99 WSIs (Austin-CRC: 53 WSIs, 226 regions, 14,385 TILs; CPTAC-COAD: 46 WSIs, 142 regions, 6,214 TILs), aiming to capture a range of regions with varying number of TILs.

To adapt SegFormer for point detection (Fig. 1C), we first converted the point annotations into a ground truth heatmap suitable for a segmentation model by creating a heatmap of 2D Gaussian distributions centred on each annotated point. This resulted in model-generated heatmaps consisting of circular areas that represent the likelihood that a pixel belongs to a TIL. Using this heatmap, we applied a blob detection algorithm to extract a series of 2D point coordinates corresponding to each detected TIL. To train the model, we used the mean squared error loss between our generated ground truth heatmaps and the predictions made by the model. Further details on our implementation are provided in the supplementary material.

Except for the loss function used, the training procedure for TIL detection was the same as TSN segmentation, but with all image data scaled to 40× magnification (Fig. 1B). At inference time, tiling was applied in the same way as for TSN segmentation. In addition, a custom point-based non-maximum suppression algorithm was applied to ensure that the same TIL detected in adjacent tiles was only counted once.

### TIL scoring

TILs were associated to tumour or stroma by looking up the coordinate in the predicted TSN mask, enabling identification of iTILs. Our iTIL density quantification approach is inspired by the existing manual approach of enumerating iTILs within five separate high-power fields (HPFs) with diameter 0.55 mm before aggregating the final count^6^. To mimic this methodology and increase robustness to potential errors in segmentation or detection, we applied our analysis in ‘local’ square fields of equivalent area, and aggregated data between fields to derive a TIL score for the slide. A field was chosen if it contained at least 10% segmented tumour, which often results in many more than five fields contributing to the final score.

To quantify iTILs in each field, we compute the total number of detected iTILs and divide them by the area occupied by tumour within that field. This metric is similar to what can be measured by a pathologist, with the exception that we normalise by area of tumour (in mm^2^). This provides a more consistent TIL measure between fields than is manually possible, as each manually assessed HPF is likely to contain varying amounts of tumour. To derive a final score for the WSI, we take the median of the per-field scores. Formally, our AI iTIL score is defined as:

$$\:{\text{m}\text{e}\text{d}\text{i}\text{a}\text{n}}_{i=0}^{F}\frac{{L}_{i}}{{T}_{i}}$$

where *L*_*i*_ is the count of iTILs in field *i*, *T*_*i*_ is the area of tumour in field *i*, and *F* is the total number of fields (potentially across multiple WSIs from the same patient).

To instil further confidence in the score produced by our method, we define a set of criteria that flags WSIs where the analysis performed may not be reliable and it is recommended that predictions should be manually inspected for full confidence. These criteria were developed to detect abnormal situations that would require further review in standard pathology practice, such as when not enough tumour tissue is available to analyse, and a separate sample would need to be assessed. Further information on the selection of flagging criteria can be found in the supplementary material. We excluded flagged cases from our survival analysis, however observed that including them did not negatively impact the survival models (supplementary material), suggesting that reasonable TIL quantifications were still being made on these cases.

### Survival analysis

We performed survival analysis on the Austin-CRC, RNSH-CRC, and MCO-CRC cohorts to evaluate the prognostic value of our AI-derived iTIL metric, and to make comparisons with available manual pathologist assessments. We applied a cut-off threshold to our iTIL metric to classify patients as high or low risk, which was identified by using the Austin-CRC cohort as a ‘discovery’ cohort, and inspecting how the stratification and hazard ratio changed as the cut-off was varied when evaluating RFS.

We validate the selected cut-off by analysing 5-year RFS in the MCO-CRC cohort (RNSH-CRC RFS data not collected), and 5-year OS in the RNSH-CRC and MCO-CRC cohorts. RFS was defined as the time from surgery to any relapse event, censored at the date of last follow-up, and OS as the time from surgery to death from any cause, censored at the date of last follow-up. To limit analysis to 5-year, any patients with a follow-up time > 1825 days had their follow-up time set to 1825 days and were censored.

We generate Kaplan-Meier curves and apply both univariate and multivariate Cox proportional hazard models to quantify the hazard ratio (HR) describing the effect of TIL scores on survival. All statistical analysis was performed in Python using the lifelines [54] package.

## Results

We present two sets of results to fully evaluate the performance of our method for assessing iTILs. First, we assess the performance of our deep learning models in each stage of our method when compared to manual pathologist annotations. Second, we report results from survival analysis performed on our discovery and validation cohorts.

### Tissue segmentation and cell detection performance

**Fig. 2:**
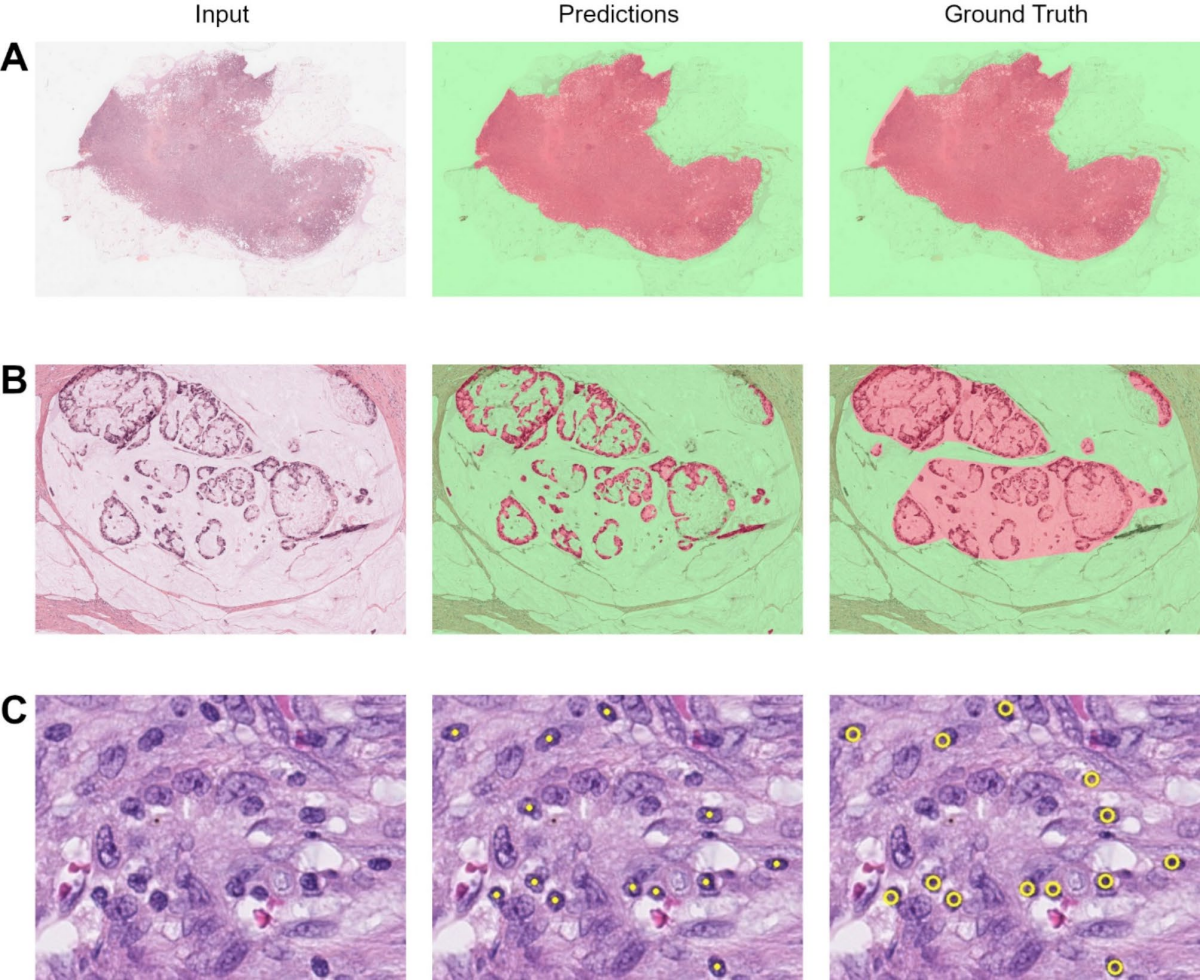
Predictions on test set regions from the broad tumour (**A**) (average F1: 0.9819), TSN (**B**) (average F1: 0.6460), and TIL (**C**) (F1: 0.9231) models. Broad tumour: green represents background and normal tissue, red represents cancerous tissue. TSN: Green represents stroma, red represents tumour

Each deep learning model was evaluated on held-out test sets before being retrained on all available data for performing survival analysis. Data splitting was performed per-patient (as opposed to per-annotated region) to ensure the models generalise across patients. Figure 2 depicts sample predictions from each model with corresponding ground truth annotations.

Evaluation of the broad tumour segmentation model across 19 WSIs from the Austin-CRC (9 WSIs) and PAIP2020 (10 WSIs) datasets showed a very high correlation between AI predictions and ground truth annotations (average F1 score: 0.9500; Supplementary Table S4).

Evaluation of the TSN model was performed across 96 regions belonging to 16 WSIs from the Austin-CRC (59 regions, 9 WSIs), and CPTAC-COAD (37 regions, 7 WSIs) datasets, with a very high concordance seen between ground truth and predictions for the tumour and stroma classes (F1: 0.8812 and 0.9101 respectively; Supplementary Table S5). Necrosis was the worst performing class (F1: 0.6407), which we attribute to the relatively small amount of annotated necrosis in our dataset (≈ 2.5% of all annotated pixels). Given necrosis is a rare occurrence compared to tumour and stroma, and our end goal is to exclude detected areas of necrosis, we consider this performance to be acceptable in the context of our method. A qualitative inspection across predictions showed generally good concordance with ground truth annotations, where some poor performing cases were attributed to imprecise annotations (Fig. 2B).

Evaluation of the TIL detection model was performed across 134 regions belonging to 42 WSIs from the Austin-CRC (31 regions, 9 WSIs) and CPTAC-COAD (103 regions, 33 WSIs) datasets. We defined TIL detections as true positives if they fell within a Euclidean distance of 4 μm from a ground truth annotation (distance chosen based on 8 μm average TIL diameter [36]). Our model achieved an average precision (AP) of 0.5163 and an F1 score of 0.5928. A qualitative inspection into model performance showed generally good concordance between ground truth and predictions. In very few examples, we noticed instances of cross-cut tumour nuclei being falsely detected as TILs, which is likely due to such examples being underrepresented in our training dataset. However, the overall impact of these tangentially sliced tissue samples is low since they are relatively rare.

To minimise both false positive and false negative detections in our TIL scoring method, we explored the application of a confidence score threshold to reject TIL detections with low confidence. A search for the optimal threshold, guided by the analysis of the F1 score across different confidence score thresholds, resulted in discarding TIL detections with a confidence score below 0.35. Further details on the selection of this confidence score are provided in the “TIL Detection Confidence Threshold Selection” section of our supplementary material.

### AI iTIL score predicts 5-Year RFS in discovery cohort

By selecting an appropriate TIL density cut-off value, our iTIL scoring method can be used to separate CRC patients into high and low risk groups. This cut-off value was selected using the Stage III Austin-CRC discovery cohort, which is independent from the datasets used for deep learning model training. Following removal of 35 flagged patients (≈ 9% of all patients), survival analysis was performed on cases from 353 patients (516 WSIs). Patients were randomly split into five equally sized groups, and survival analysis was performed five times, with a different group excluded from analysis each time. We systematically inspected how changes to the chosen binary cut-off impacted RFS stratification in terms of hazard ratio and significance and selected the threshold that resulted in the most significant stratification of the group. This search yielded 17 iTILs per-mm^2^ tumour area as the optimal cut-off within each survival analysis performed. This cut-off was fixed for subsequent survival analysis on all cohorts.

We performed survival analysis in terms of 5-Year RFS on the entire Austin-CRC cohort using 17 as the cut-off threshold for stratifying patients by risk. Manual iTIL scores from pathologist DW were available using the prior method [6], where patients with ≥ 2 TILs per-HPF (averaged across 5 HPFs) were deemed TIL-High, otherwise TIL-Low. Better stratification was observed when using the AI iTIL score (HR 2.05, 95% CI 1.46–2.88; *p* < 0.0001) compared with the manual pathologist score (HR 1.75, 95% CI 1.10–2.78; *p* = 0.0186) (Supplementary Figure S8). The AI iTIL score remained significant in a multivariate analysis (Table 1).

**Table 1:** Univariate and multivariate results for Austin-CRC, combined RNSH-CRC + MCO-CRC, and RNSH-CRC cohorts. Adjuvant chemotherapy use was not collected for the RNSH-CRC cohort. *p < 0.05

Austin-CRC	Univariate				Multivariate			
5-Year RFS	*n*	HR	95% CI	*p*	*n*	HR	95% CI	*p*
Age (Decades)	353	1.07	0.93–1.22	0.349	266	1.16	0.97–1.39	0.100
Gender (Female vs. Male)	353	0.69	0.49–0.97	0.032*	266	0.77	0.53–1.14	0.190
Site (Proximal vs. Distal)	353	0.88	0.63–1.23	0.442	266	0.95	0.61–1.47	0.811
T Stage (4 vs. 1–3)	353	2.38	1.69–3.35	< 0.001*	266	1.90	1.28–2.83	0.001*
N Stage (2 vs. 0–1)	353	1.68	1.19–2.36	0.003*	266	1.88	1.24–2.85	0.003*
Nodes Examined (< 12 vs. 12+)	324	1.77	1.17–2.68	0.007*	266	1.79	1.10–2.91	0.018*
Adjuvant Chemotherapy (yes vs. no)	309	0.92	0.63–1.34	0.664	266	0.97	0.60–1.57	0.898
Grade (high vs. low)	350	1.43	1.01–2.03	0.046*	266	1.11	0.70–1.74	0.662
Lymphovascular Invasion (yes vs. no)	353	1.42	1.02–1.99	0.040*	266	1.07	0.73–1.58	0.732
Extramural Venous Invasion (yes vs. no)	353	1.55	1.08–2.21	0.016*	266	1.29	0.85–1.96	0.227
MMR Status (pMMR vs. dMMR)	335	1.61	0.95–2.72	0.074	266	0.77	0.39–1.49	0.431
AI iTIL Risk (high vs. low)	353	2.05	1.46–2.88	< 0.001*	266	2.03	1.35–3.05	< 0.001*
**RNSH-CRC + MCO-CRC**								
**5-Year OS**	**n**	**HR**	**95% CI**	**p**	**n**	**HR**	**95% CI**	**p**
Age (Decades)	1955	1.40	1.29–1.51	< 0.001*	1901	1.51	1.38–1.65	< 0.001*
Gender (Female vs. Male)	1955	0.87	0.73–1.04	0.135	1901	0.80	0.67–0.96	0.016*
Site (Proximal vs. Distal)	1945	1.04	0.87–1.25	0.632	1901	0.98	0.80–1.20	0.861
Stage (III vs. II)	1955	2.31	1.92–2.79	< 0.001*	1901	1.66	1.32–2.08	< 0.001*
T Stage (4 vs. 1–3)	1955	2.39	2.00-2.86	< 0.001*	1901	1.87	1.55–2.27	< 0.001*
N Stage (2 vs. 0–1)	1954	2.56	2.10–3.13	< 0.001*	1901	1.80	1.42–2.29	< 0.001*
Nodes Examined (< 12 vs. 12+)	1954	1.71	1.40–2.10	< 0.001*	1901	1.70	1.37–2.10	< 0.001*
Grade (high vs. low)	1944	1.79	1.49–2.15	< 0.001*	1901	1.31	1.07–1.60	0.009*
Lymphovascular Invasion (yes vs. no)	1945	2.16	1.81–2.58	< 0.001*	1901	1.28	1.03–1.60	0.026*
Extramural Venous Invasion (yes vs. no)	1926	1.91	1.58–2.30	< 0.001*	1901	1.26	1.01–1.57	0.039*
MMR Status (pMMR vs. dMMR)	1951	1.13	0.90–1.42	0.285	1901	1.03	0.79–1.34	0.828
AI iTIL Risk (high vs. low)	1955	1.67	1.39-2.00	< 0.001*	1901	1.37	1.13–1.66	0.001*
**RNSH-CRC**								
**5-Year OS**	**n**	**HR**	**95% CI**	**p**	**n**	**HR**	**95% CI**	**p**
Age (Decades)	1070	1.37	1.23–1.53	< 0.001*	1045	1.50	1.33–1.71	< 0.001*
Gender (Female vs. Male)	1070	1.13	0.88–1.46	0.327	1045	0.98	0.76–1.28	0.902
Site (Proximal vs. Distal)	1063	1.35	1.04–1.74	0.022*	1045	1.22	0.92–1.61	0.168
Stage (III vs. II)	1070	2.76	2.09–3.64	< 0.001*	1045	1.71	1.21–2.42	0.002*
T Stage (4 vs. 1–3)	1070	2.63	2.05–3.38	< 0.001*	1045	1.90	1.45–2.48	< 0.001*
N Stage (2 vs. 0–1)	1069	3.15	2.40–4.14	< 0.001*	1045	2.23	1.61–3.10	< 0.001*
Nodes Examined (< 12 vs. 12+)	1069	1.49	1.07–2.07	0.017*	1045	1.58	1.12–2.22	0.010*
Grade (high vs. low)	1059	1.98	1.53–2.55	< 0.001*	1045	1.34	1.01–1.77	0.042*
Lymphovascular Invasion (yes vs. no)	1068	2.52	1.96–3.24	< 0.001*	1045	1.24	0.92–1.68	0.164
Extramural Venous Invasion (yes vs. no)	1067	2.02	1.56–2.62	< 0.001*	1045	1.41	1.06–1.87	0.019*
MMR Status (pMMR vs. dMMR)	1066	1.13	0.84–1.54	0.421	1045	1.13	0.79–1.61	0.510
AI iTIL Risk (high vs. low)	1070	1.86	1.45–2.39	< 0.001*	1045	1.48	1.13–1.93	0.004*

Assuming in practice that a manually assessed HPF would contain approximately 40– 60% tumour, an equivalent measure of 2 TILs per-HPF in TILs per-mm^2^ would be in the range of 14–21 (with 17 TILs corresponding to approximately 50% tumour in a HPF). This suggests that the manual and AI iTIL thresholds used are similar.

### AI iTIL score predicts 5-Year RFS and 5-Year OS in validation cohorts

To evaluate the prognostic power of our iTIL scoring method and test robustness of the identified iTIL cut-off, we applied our method to two validation cohorts that were not used during deep learning model development or metric cut-off search. Following removal of flagged cases, survival analysis was performed on 1070 patients from the RNSH-CRC cohort (excluding 38 flagged patients, ≈ 3.4%), and 885 patients from the MCO-CRC cohort (excluding 53 flagged patients, ≈ 5.7%), using the iTIL cut-off of 17 per-mm^2^ tumour to stratify by risk.

**Fig. 3:**
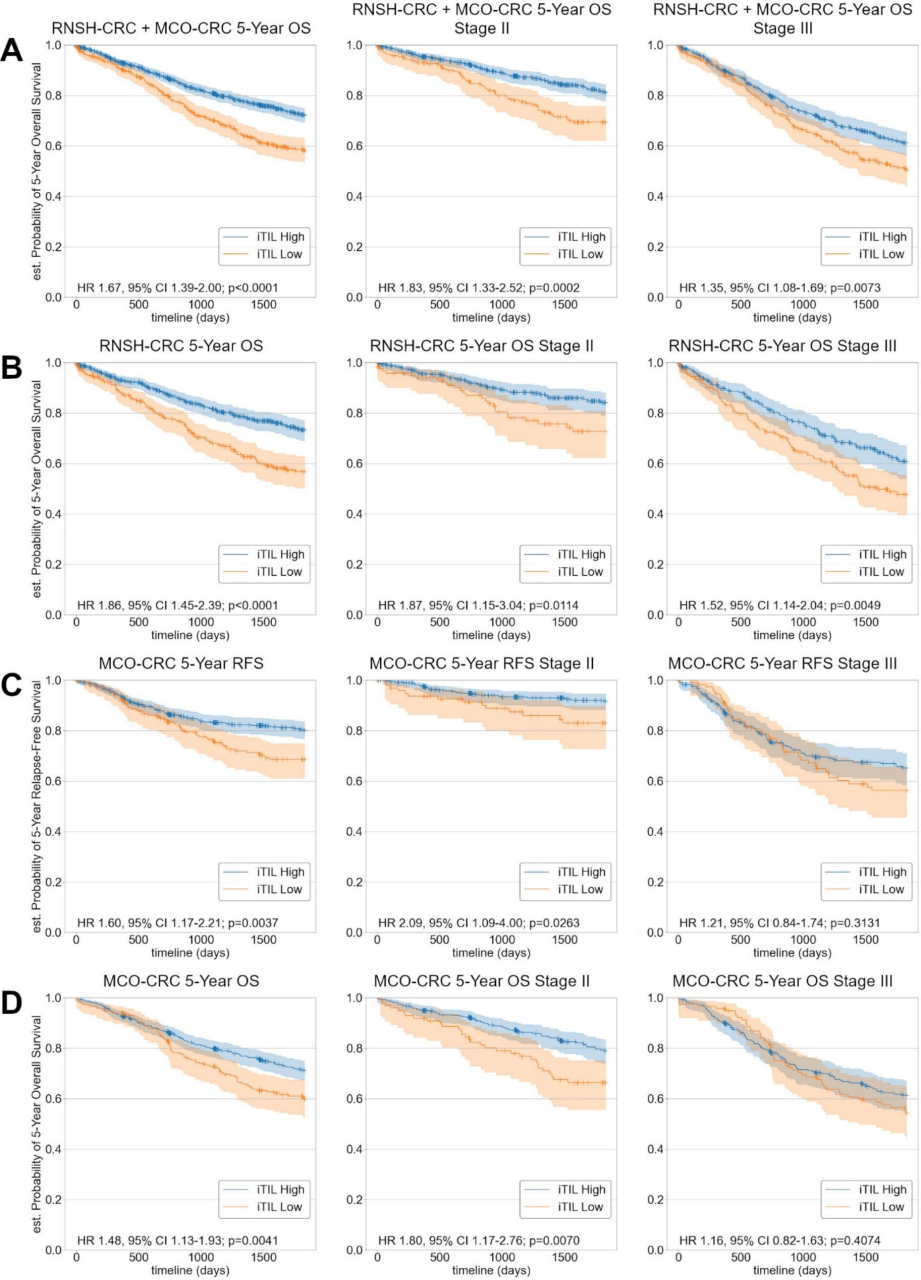
Kaplan-Meier curves illustrating stratification by the AI-generated iTIL score. (**A**) Combined cohort 5-Year OS, (**B**) RNSH-CRC 5-Year OS, (**C**) MCO-CRC 5-Year RFS, (**D**) MCO-CRC 5-Year OS

The AI iTIL score significantly stratified the combined RNSH-CRC + MCO-CRC cohort in terms of 5-Year OS (Table 1), stratifying both Stage II (HR 1.93, 95% CI 1.33–2.52; *p* = 0.0002) and III (HR 1.35, 95% CI 1.08–1.69; *p* = 0.0073) cases independently (Fig. 3A).

Significant stratification by the AI iTIL score was achieved in the RNSH-CRC cohort in terms of 5-Year OS in a univariate analysis (HR 1.86, 95% CI 1.45–2.39; *p* < 0.0001) and was shown to stratify Stage II and III cases independently (Fig. 3B). The AI iTIL score remained significant in a multivariate analysis (HR 1.48, 95% CI 1.13–1.93; *p* = 0.004, Table 1).

In a univariate analysis, significant stratification of the entire MCO-CRC cohort and Stage II cases was achieved in terms of both 5-Year RFS and 5-Year OS (Fig. 3C and D), though not for Stage III cases. We observed the available manual assessments significantly stratified 5-Year RFS in this cohort (for both Stage II and III), however not 5-Year OS (HR 1.50, 95% CI 0.96–2.34; *p* = 0.0728, and HR 1.49, 95% CI 0.96–2.31; *p* = 0.0737 for Stage II and III respectively). The AI iTIL score was not significant in a multivariate analysis (Supplementary Table S6), though we note that a prior study quantifying TILs with deep learning in the MCO-CRC cohort [30] was also unable to achieve significance in a multivariate analysis.

### AI iTIL score stratifies stage II clinical high risk

A potential application of our iTIL score is to help predict Stage II CRC patients with poorer prognosis, and thus aid in deciding which patients should be treated with adjuvant chemotherapy. Adjuvant chemotherapy is often used to treat clinical high-risk Stage II CRC patients, due to their poorer prognosis, whereas clinical low-risk Stage II CRC patients are typically not treated with chemotherapy. We therefore sought to investigate whether our AI iTIL score can further stratify patients with clinical high or low risk features. Clinical high risk in Stage II CRC was defined as having either T4, high grade, extramural venous invasion, lymphovascular invasion, or fewer than 12 lymph nodes removed, whilst low risk was defined as having none of those features [55]. Supplementary Table S7 shows a breakdown of clinical high and low risk patients in each cohort.

**Fig. 4:**
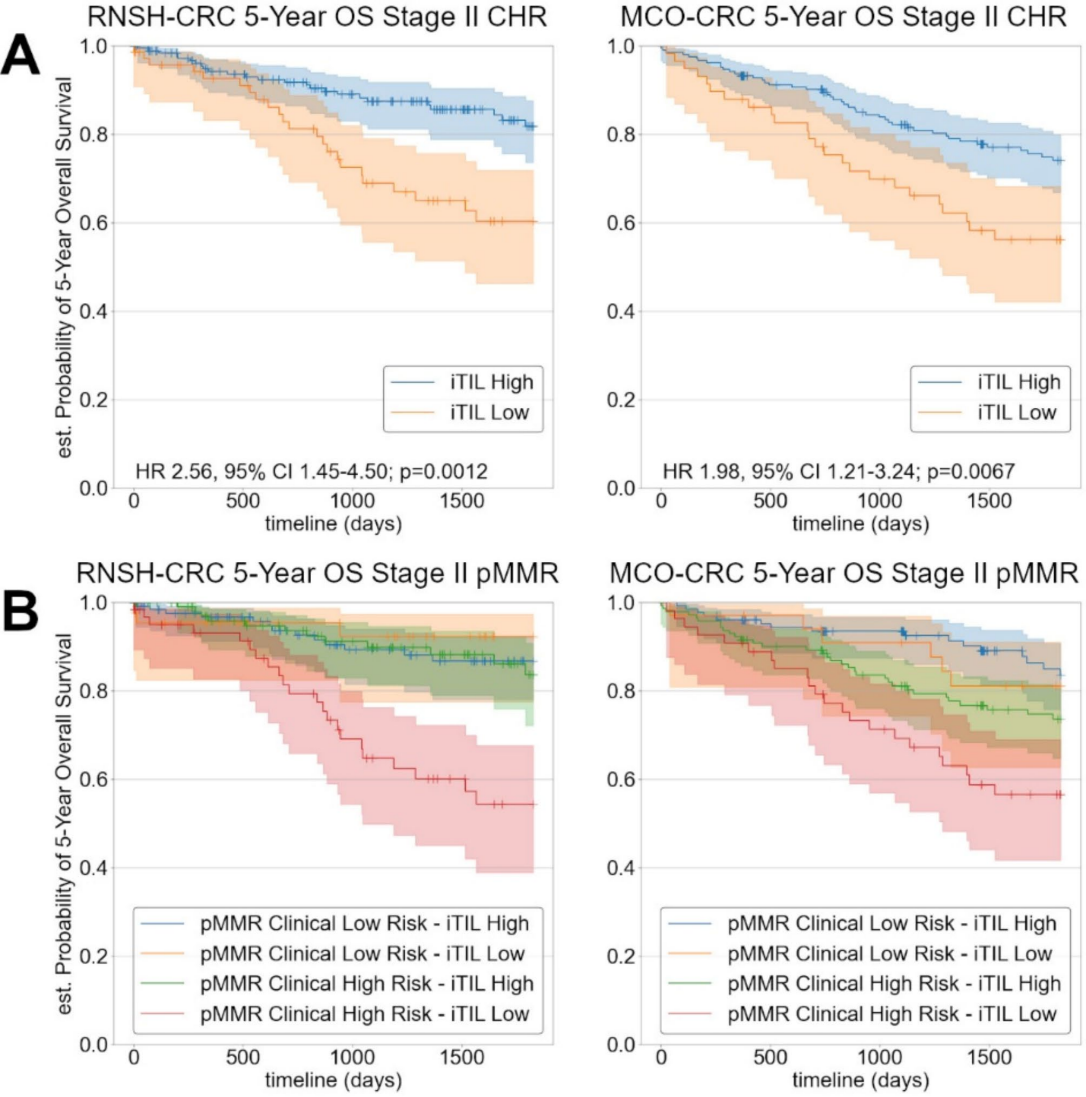
Kaplan-Meier curves illustrating stratification of 5-Year OS by the AI iTIL score in Stage II clinical high risk cases (**A**), and Stage II pMMR clinical high and low risk cases (**B**) in each of the validation cohorts. Significant stratification, particularly in the pMMR clinical high risk group, suggests the AI iTIL score could be used as an additional signal to identify patients with better/worse outcomes

We found significant stratification by our AI iTIL score for Stage II clinical high risk patients in each of our validation cohorts in both 5-Year RFS (MCO-CRC HR 2.32, 95% CI 1.10–4.92; *p* = 0.0277) and 5-Year OS (Fig. 4A).

Further inspecting this group, we identified that our AI iTIL score was able to stratify proficient MMR (pMMR) Stage II clinical high risk patients, with identified TIL-High patients having an outcome approaching pMMR clinical low risk patients, and TIL-Low patients having significantly worse outcomes (RNSH-CRC: HR 3.65, 95% CI 1.80–7.37; *p* = 0.0003; MCO-CRC: HR 1.83, 95% CI 1.07–3.15; *p* = 0.0275). The AI iTIL score did not further stratify pMMR clinical low risk patients (Fig. 4B).

To further validate these results, we performed a combined cohort-stratified multivariate analysis in terms of 5-Year OS for both Stage II clinical high risk, and Stage II pMMR clinical high risk (Supplementary Tables S8 and S9). The AI iTIL score remained significant in both analyses (HR 2.00 95% CI 1.34–2.96; *p* < 0.001, and HR 2.17, 95% CI 1.42–3.33; *p* < 0.001 respectively).

Whilst these results are promising, a further validation study in Stage II CRC is needed to investigate whether our AI iTIL score can assist in treatment planning.

## Discussion

The presence of TILs is known to be prognostic in CRC, having shown links to improved patient survival [4, 6–8]. Despite the potential to inform patient treatment, TIL assessments are yet to be incorporated into CRC clinical practice, partly due to lack of consensus regarding methodology, interobserver variability in manual estimation methods, and the time taken for a manual assessment to be made.

Automated systems can be leveraged to address these limitations, however it is important that their outputs are interpretable, so pathologists can explain how an assessment was derived. In this work we have described a novel automated TIL detection method that enables the quantification of iTILs in H&E-stained CRC samples, with outputs of our method designed for interpretability. This was achieved by fusing results from three separate deep learning models that: segment cancerous tissue from normal tissue and background; separate tumour, stroma, and necrosis within cancerous tissue; and detect TILs within cancerous tissue.

There are few existing works that utilise deep learning to quantify TILs in H&E-stained CRC samples [27–31], however our work is different in a few ways. First, our approach is entirely automated, requiring no intervention by pathologists, except to inspect the calls made by our method. Scores are still produced for cases flagged for review, and we observed that it is relatively uncommon, with only ≈ 5% of all cases analysed being flagged. Second, we do not perform classification at the patch-level, but instead at the pixel-level. The outputs of our method are granular and can be directly interpreted. This extra resolution helps to ensure derived TIL scores are more accurate, given there is no ambiguity in what is contained in a single patch. Third, our method analyses all cancerous tissue in the slide in a computationally efficient way, such that we can generate results in a timely manner, whilst being able to analyse a large amount of data and extract metrics derived from analysing the entire slide. The median number of tiles sampled per WSI was 424, 399, and 264 on the Austin-CRC, RNSH-CRC, and MCO-CRC cohorts respectively, which far exceeds the 5 random fields sampled according to the prior manual assessment method. Fourth, our approach in segmenting tumour and stromal regions enables TIL density within either compartment to be assessed. The International TILs Working Group method for assessing stromal TILs in breast cancer was previously reported as significant in CRC [8], so there is potential to evaluate if we can reproduce these results from our method, and test whether a combined iTIL and stromal TIL approach may be a more powerful predictor.

Analysing the performance of each deep learning model showed a high concordance between model predictions and pathologist annotations, verified both quantitatively and qualitatively. Two limitations were noted in our current design that we plan to address in future work. First, we observed instances of tangentially sliced tumour nuclei being falsely detected as TILs. We suspect this is primarily due to the model not having seen many of these examples during training, and therefore has not learned to distinguish them reliably. We plan to directly address this limitation in future work by updating our training set to contain more of these examples. Second, our model does not distinguish mucin from stroma, which typically leads to mucin being predicted as stroma. This is not problematic for our current scoring method, as stromal areas are not assessed, however we plan to add capacity for our model to segment mucin as we explore stromal-related metrics in the future.

Using the Austin-CRC cohort, we were able to identify a binary cut-off threshold for our iTIL metric to classify patients by risk, which demonstrated generalisation to two independent validation cohorts. Given our deep learning models were not trained on data used for survival analysis in the Austin- CRC cohort, this also validates that our deep learning models can generalise to unseen data.

When evaluated in the two validation cohorts, we found our AI iTIL score significantly stratified both cohorts in combined and individual analysis, with the exception of Stage III cases in the MCO-CRC cohort. Generally, stratification of Stage II patients was more significant than for Stage III. For future work we plan to explore whether our method could assist in treatment planning for Stage II CRC by performing a study on a Stage II CRC cohort. In a multivariate analysis, the AI iTIL score was significant in the combined cohort and RNSH-CRC cohort analyses, however not for MCO-CRC. Inspecting distributions of the continuous valued AI iTIL score, we found that generally Stage II cases had higher TIL counts than Stage III (Supplementary Figure S9). Given outcome is generally better in earlier stages, this finding is consistent with higher TIL counts contributing to better patient outcome. We also found that the median detected iTIL count in Stage III MCO-CRC was higher than for other cohorts, which may explain why significant stratification was not achieved in MCO-CRC Stage III. We plan to investigate performance on the MCO-CRC dataset further as part of future work.

An important finding was the ability of our AI iTIL score to stratify Stage II clinical high risk patients, and particularly Stage II pMMR clinical high risk cases, where an interest lies in identifying the most at-risk patients. Further validation of our method in a Stage II cohort is required to fully understand the implication of this finding. We found TIL-High cases had outcomes approaching Stage II pMMR clinical low risk cases, and TIL-Low cases had significantly poorer outcomes. In the RNSH-CRC cohort, the survival curve of TIL-High cases overlapped with pMMR clinical low risk. These findings were significant in both univariate and multivariate analyses.

## Conclusions

Our results show that our fully automated method is able to quantify iTIL density in H&E-stained CRC WSIs, with demonstrated capability to generalise to large independent patient cohorts from different centres. Our method addresses limitations faced by manual scoring in that it evaluates the entire tumour region, is repeatable, does not suffer from intra- or inter-rater variability, has potential to be applied at scale, and has built-in safeguards to flag difficult cases. It also addresses limitations faced by other automated approaches in that it has been evaluated in multiple large patient cohorts from different centres with no further tuning, and identifies tumour regions and TILs at the most granular level, giving it the ability to provide a more accurate measure of iTIL density. We anticipate that this method can serve as an additional prognostic tool for pathologists and oncologists, with the ability for them to inspect predictions made by individual models to validate the plausibility of the derived iTIL score.

## Electronic supplementary material

Below is the link to the electronic supplementary material.

Supplementary Material 1

## Data Availability

Access to private data used for deep learning model training and survival analysis requires approval by data custodians. Requests can be made to the corresponding author for further information on who to contact for access. Public data used for deep learning model training can be accessed online. Model training and TIL scoring code is planned to be released in the future (model weights can be provided if access to private data is granted).

## Footnotes

1 https://paip2020.grand-challenge.org/Dataset/

2 Data used in this publication were generated by the National Cancer Institute Clinical Proteomic Tumour Analysis Consortium (CPTAC).

3 UNSW researchers bear no responsibility for the further analysis or interpretation of the MCO data.

## Abbreviations

CRC: Colorectal cancer
H&E: Haematoxylin and Eosin
HPF: High-power field
HR: Hazard ratio
IHC: Immunohistochemistry
iTILs: Intraepithelial tumour-infiltrating lymphocytes
MMR: Mismatch repair
OS: Overall survival
RFS: Relapse-free survival
TILs: Tumour-infiltrating lymphocytes
TNM: Tumour, node, metastasis
TSN: Tumour, stroma, necrosis
WSI: Whole slide image
