# Supplementary material for: Automated deep learning-based assessment of tumour-infiltrating lymphocyte density determines prognosis in colorectal cancer
Source: J Transl Med. 2025 Mar 10;23:298. doi: 10.1186/s12967-025-06254-3 (PMC11892243; doi:10.1186/s12967-025-06254-3)
Supplement: Supplementary file 1 — Supplementary Material 1 [file 12967_2025_6254_MOESM1_ESM.docx]

**Supplementary Material**

**Comparison to Related Works**


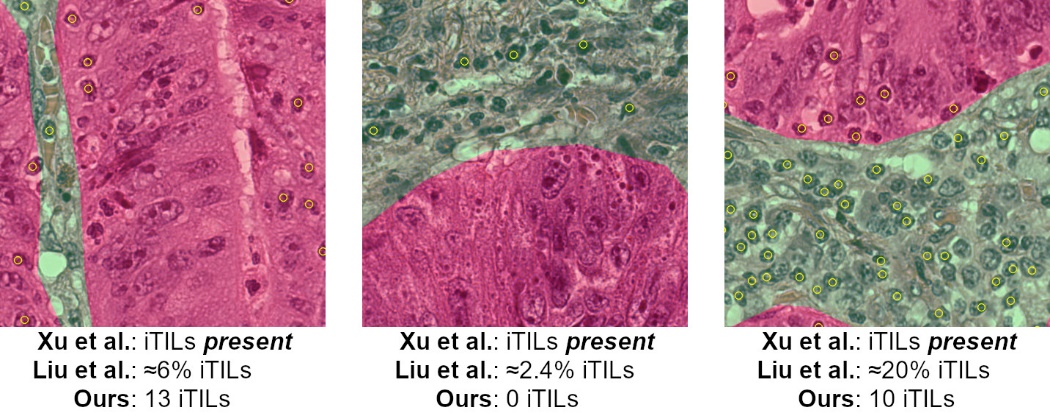


Figure S1: Example 112 μm^2^ tissue patches annotated by pathologist DW, of the same area used for tumour^30^ and TIL^29^ localisation, with estimations on what each method would predict. Green overlay represents stroma, red overlay represents tumour, yellow circles represent lymphocytes. Whilst patch-based classification for tumour localisation works reasonably well when patches predominantly contain tumour, it has limitations for patches that contain multiple tissue types (e.g. tumour and stroma). Ambiguity is also introduced where it is unclear how much tumour needs to be present for a patch to be classified as tumour. This has the consequence that stromal TILs can be included as part of the iTIL density estimation, resulting in situations where iTILs can be overcalled. Patch-based TIL detection^29^ has the added deficiency that there is no way to distinguish between patches that contain few or many TILs. These limitations are apparent in the second example, where no iTILs are present, but both patch-based approaches detect a presence of iTILs.

**Dataset Breakdown**


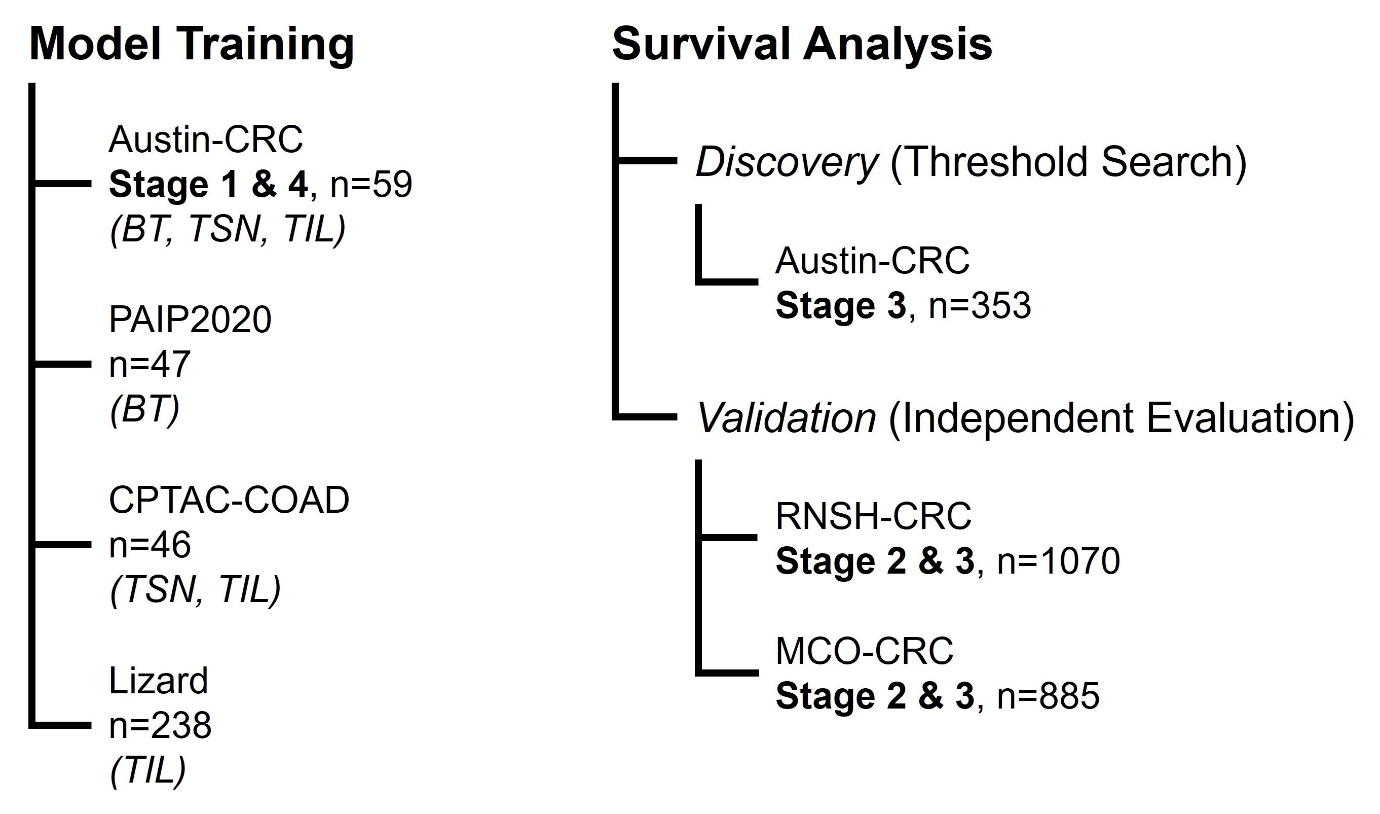


Figure S2: Tree diagram illustrating the breakdown of datasets used for model training and survival analysis. For model training, *n* refers to the number of WSIs in the dataset, each with one or more annotated region. For survival analysis, *n* represents the number of patients included from each dataset. Bracketed terms associated with the model training datasets indicate the models trained using that dataset. BT: Broad Tumour segmentation model, TSN: Tumour/Stroma/Necrosis segmentation model, TIL: TIL detection model.

**Clinicopathological Features**

|  | **Austin-CRC** | | | | **RNSH-CRC** | | | | **MCO-CRC** | | | |
| --- | --- | --- | --- | --- | --- | --- | --- | --- | --- | --- | --- | --- |
|  |  | TIL-Low | TIL-High |  |  | TIL-Low | TIL-High |  |  | TIL-Low | TIL-High |  |
|  | n | n=148 (41.9) | n=205 (58.1) | p | n | n=333 (31.1) | n=737 (68.9) | p | n | n=215 (24.3) | n=670 (75.7) | p |
| Age (years) | 353 |  |  |  | 1070 |  |  |  | 885 |  |  |  |
| Mean ± SD |  | 67.3 ± 12.5 | 69.3 ± 13.2 | 0.164 |  | 71.3 ± 13.6 | 72.2 ± 13.4 | 0.295 |  | 69.1 ± 12.4 | 69.7 ± 12.4 | 0.520 |
| Median |  | 69.2 | 71.0 |  |  | 73.0 | 75.0 |  |  | 70.0 | 71.0 |  |
| Range |  | 28.9-91.0 | 22.0-94.0 |  |  | 28.0-100.0 | 27.0-101.0 |  |  | 24.0-97.0 | 27.0-99.0 |  |
| Gender | 353 |  |  |  | 1070 |  |  |  | 885 |  |  |  |
| Female |  | 72 (48.6) | 116 (56.6) | 0.172 |  | 149 (44.7) | 405 (55.0) | 0.002* |  | 97 (45.1) | 319 (47.6) | 0.576 |
| Male |  | 76 (51.4) | 89 (43.4) |  |  | 184 (55.3) | 332 (45.0) |  |  | 118 (54.9) | 351 (52.4) |  |
| Site | 353 |  |  |  | 1063 |  |  |  | 882 |  |  |  |
| Proximal |  | 64 (43.2) | 127 (62.0) | <0.001* |  | 164 (49.5) | 433 (59.2) | 0.004* |  | 73 (34.1) | 280 (41.9) | 0.051 |
| Distal |  | 84 (56.8) | 78 (38.0) |  |  | 167 (50.5) | 299 (40.8) |  |  | 141 (65.9) | 388 (58.1) |  |
| Stage | 353 |  |  |  | 1070 |  |  |  | 885 |  |  |  |
| II |  | 0 (0.0) | 0 (0.0) |  |  | 127 (38.1) | 408 (55.4) | <0.001* |  | 99 (46.0) | 379 (56.6) | 0.009* |
| III |  | 148 (100.0) | 205 (100.0) |  |  | 206 (61.9) | 329 (44.6) |  |  | 116 (54.0) | 291 (43.4) |  |
| Grade | 350 |  |  |  | 1059 |  |  |  | 885 |  |  |  |
| Low |  | 105 (71.4) | 130 (64.0) | 0.181 |  | 239 (72.4) | 518 (71.1) | 0.702 |  | 169 (78.6) | 498 (74.3) | 0.240 |
| High |  | 42 (28.6) | 73 (36.0) |  |  | 91 (27.6) | 211 (28.9) |  |  | 46 (21.4) | 172 (25.7) |  |
| Mucinous | 350 |  |  |  | 1070 |  |  |  | 885 |  |  |  |
| No |  | 97 (66.0) | 135 (66.5) | 1.000 |  | 298 (89.5) | 676 (91.7) | 0.285 |  | 182 (84.7) | 517 (77.2) | 0.025* |
| Yes |  | 50 (34.0) | 68 (33.5) |  |  | 35 (10.5) | 61 (8.3) |  |  | 33 (15.3) | 153 (22.8) |  |
| T Stage | 353 |  |  |  | 1070 |  |  |  | 885 |  |  |  |
| 1-3 |  | 101 (68.2) | 148 (72.2) | 0.493 |  | 199 (59.8) | 570 (77.3) | <0.001* |  | 153 (71.2) | 532 (79.4) | 0.016* |
| 4 |  | 47 (31.8) | 57 (27.8) |  |  | 134 (40.2) | 167 (22.7) |  |  | 62 (28.8) | 138 (20.6) |  |
| N Stage | 353 |  |  |  | 1069 |  |  |  | 885 |  |  |  |
| 0-1 |  | 98 (66.2) | 137 (66.8) | 0.995 |  | 269 (80.8) | 650 (88.3) | 0.001* |  | 173 (80.5) | 577 (86.1) | 0.058 |
| 2 |  | 50 (33.8) | 68 (33.2) |  |  | 64 (19.2) | 86 (11.7) |  |  | 42 (19.5) | 93 (13.9) |  |
| Extramural Venous Invasion | 353 |  |  |  | 1067 |  |  |  | 859 |  |  |  |
| No |  | 107 (72.3) | 151 (73.7) | 0.871 |  | 232 (69.9) | 586 (79.7) | <0.001* |  | 146 (69.9) | 503 (77.4) | 0.035* |
| Yes |  | 41 (27.7) | 54 (26.3) |  |  | 100 (30.1) | 149 (20.3) |  |  | 63 (30.1) | 147 (22.6) |  |
| Lymphovascular Invasion | 353 |  |  |  | 1068 |  |  |  | 877 |  |  |  |
| No |  | 74 (50.0) | 110 (53.7) | 0.568 |  | 180 (54.1) | 475 (64.6) | 0.001* |  | 117 (54.9) | 424 (63.9) | 0.024* |
| Yes |  | 74 (50.0) | 95 (46.3) |  |  | 153 (45.9) | 260 (35.4) |  |  | 96 (45.1) | 240 (36.1) |  |
| MMR Status | 335 |  |  |  | 1066 |  |  |  | 885 |  |  |  |
| pMMR |  | 133 (93.0) | 141 (73.4) | <0.001* |  | 296 (89.4) | 519 (70.6) | <0.001* |  | 203 (94.4) | 524 (78.2) | <0.001* |
| dMMR |  | 10 (7.0) | 51 (26.6) |  |  | 35 (10.6) | 216 (29.4) |  |  | 12 (5.6) | 146 (21.8) |  |
| BRAF Status | 314 |  |  |  | 1063 |  |  |  | 881 |  |  |  |
| Wild type |  | 118 (86.1) | 128 (72.3) | 0.005* |  | 266 (80.6) | 516 (70.4) | <0.001* |  | 200 (94.3) | 555 (83.0) | <0.001* |
| Mutated |  | 19 (13.9) | 49 (27.7) |  |  | 64 (19.4) | 217 (29.6) |  |  | 12 (5.7) | 114 (17.0) |  |
| Adjuvant Chemotherapy | 309 |  |  |  | N/A |  |  |  | 867 |  |  |  |
| No |  | 35 (26.3) | 64 (36.4) | 0.080 |  | N/A | N/A |  |  | 100 (47.8) | 408 (62.0) | <0.001* |
| Yes |  | 98 (73.7) | 112 (63.6) |  |  | N/A | N/A |  |  | 109 (52.2) | 250 (38.0) |  |

Table S1: Clinicopathological features of the three independent cohorts used for survival analysis. Percentages for columns shown in round brackets. *p<0.05, calculated using t-test (age), otherwise chi-square test.

**Model Training and Inference Details**

*Hyperparameters*

When training each model, an initial hyperparameter search was conducted by randomly splitting the available annotated model training data into *train/val/test* subsets with an approximate 70%/10%/20% distribution. To prevent contamination across subsets, all data from a single patient was assigned to a single subset. The F1 score achieved on the *val* subset was used to identify the hyperparameters that achieved the best performance. After selecting the optimal hyperparameters, each model was retrained using the full model training dataset.

The loss functions used in training include Cross Entropy and Mean Squared Error (MSE). The Cross Entropy loss is given by:

$$Cross Entropy=-\frac{1}{N}\sum_{i=1}^{N} \log\left( \frac{e^{z_{i,c*}}}{\sum_{k=1}^{C} e^{z_{i,k}}} \right)$$

where *N* is the total number of pixels in the batch, *i* represents a single pixel, *C* is the total number of classes in the segmentation task, $z_{i,k}$ represents the logit predicted by the model for class *k* at pixel *i*, and *c** is the ground truth class for pixel *i*.

The MSE loss is given by:

$$MSE=\frac{1}{N}\sum_{i=1}^{N} {(y_{i}-\hat{y}_{i})}^{2}$$

where $y_{i}$ represents the ground truth and $\hat{y}_{i}$ represents the predicted value. Table S2 summarises the final hyperparameters used for each model.

| **Hyperparameter** | **Broad Tumour Segmentation** | **Tumour/Stroma/Necrosis Segmentation** | **TIL Detection** |
| --- | --- | --- | --- |
| Model | SegFormer-B0 | SegFormer-B0 | SegFormer-B1 |
| MPP | 4 | 0.5 | 0.25 |
| Epochs | 500 | 2000 | 2000 |
| Learning Rate | 0.00005 | 0.00006 | 0.00006 |
| Learning Rate Scheduler | Linear Decay | Linear Decay | Linear Decay |
| Optimiser | AdamW | AdamW | AdamW |
| Loss Function | Cross Entropy | Cross Entropy | MSE |

Table S2: Hyperparameters used when training each model.

*Data Augmentation*

To improve model robustness, various forms of data augmentation were applied during training for each of the three models. These included cropping, rotation, scaling, flipping, Gaussian blurring, colour jittering, and downscaling. Augmentations were implemented by first taking a random 512×512px crop at the specified MPP from an annotated region for each model. A random rotation angle between -180° and 180° was then applied. To prevent empty pixel data from appearing after rotation, a larger initial crop was taken, followed by a secondary crop to ensure a final 512×512px region.

All other augmentations were applied with a 50% probability. When scaling was applied, the scale factor was sampled randomly between 0.9 and 1.1. For Gaussian blurring, the kernel size was chosen randomly from 3, 5, or 7. Colour jittering was applied to brightness, contrast, and saturation (with factors between 0.9 and 1.1) and to hue (with factors between 0.95 and 1.05). Downscaling was performed with a scale factor of 0.25 to simulate lower-resolution data.

*Handling Tiling Artefacts at Inference Time*

A downside to naïve tiling is that each tile misses contextual information at its boundaries. To overcome this, we define an ‘output crop margin’ from the tile border. When running inference on a tile, we discard predictions made within the output crop margin, based on the assumption that such predictions do not have enough context to be reliable. To ensure predictions cover the entire WSI without gaps, we perform inference on overlapping tiles. For example, using a 96px output crop margin, predictions are only retained for the inner 320×320px of a 512×512px tile.

We use a 96px, 64px, and 128px output crop margin for each of our broad tumour segmentation, TSN segmentation, and TIL detection models respectively.


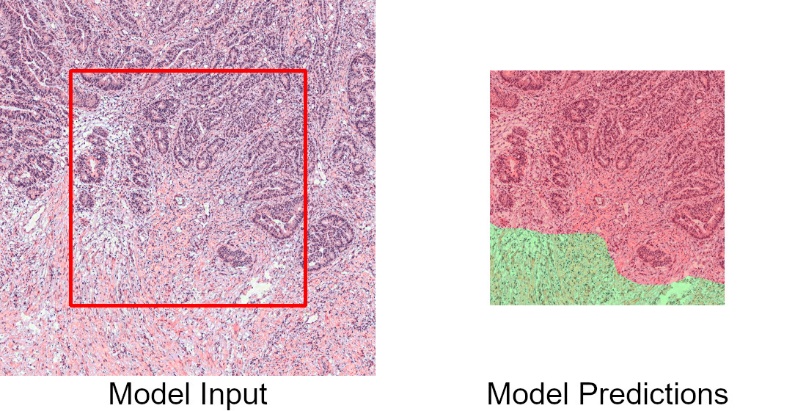


Figure S3: An illustration of the output crop margin applied for the broad tumour segmentation model. Given a 512×512px input tile, predictions are only retained for the inner area (red box) within a given margin (‘output crop margin’) of the border. For the broad tumour model, the output crop margin was 96px, meaning given a 512×512px input area, only predictions for the inner 320×320px were retained. Red overlay corresponds to predicted broad tumour, whilst green corresponds to normal tissue or background.

*TSN Tiling Algorithm*

Given that TSN segmentation should only be applied in areas of cancerous tissue, a modified version of the WSI tiling algorithm used for broad tumour segmentation was employed to generate the TSN mask. Instead of densely tiling the entire WSI, we generated a set of tiles containing at least 75% broad tumour foreground and applied TSN segmentation only within those tiles. This was done by scanning through the broad tumour mask and creating tiles, at the output size, that contained sufficient foreground.

**TIL Detection Dense Prediction Model**

The output of a typical segmentation network for an image in a batch has dimensionality *(N,H,W)*, where *N* represents the number of classes being predicted, and *H* and *W* represent the height and width of the area that segmentation is being performed in. In other words, the model outputs *N* heatmaps, one for each class, where each pixel in each heatmap is a logit that correlates to the degree of confidence the model places on the pixel belonging to that class.

To use a SegFormer model for point detection, we generate a ground truth ‘heatmap’ that contains 2D Gaussian distributions centred on each ground truth point, with every value in the heatmap bound between [0, 1]. The equation used to generate the 2D Gaussian distribution is given by:

$$f\left( x,y \right)=exp\left( -\frac{\left( x-x_{0} \right)^{2}+\left( y-y_{0} \right)^{2}}{2\sigma^{2}} \right)$$

where *x_0_,y_0_* are the coordinates of the centre of the Gaussian, corresponding to the location of the ground truth point, and *σ* is the standard deviation. We set the Gaussian’s standard deviation to approximately 1.14µm, such that seven standard deviations span 8µm, encompassing the majority of each corresponding TIL. The dimensionality of this heatmap matches the output: *(N,H,W)* (Figure S4).

Training loss is computed by first applying a Sigmoid to the segmentation output logits, bounding them to [0, 1]:

$$\sigma\left( x \right)=\frac{1}{1+e^{-x}}$$

Then, the MSE is computed between the model output and the ground truth heatmap.

At inference time, per-class point detections are extracted by applying a Sigmoid to the segmentation output logits, followed by Gaussian modulation and then performing blob detection. Gaussian modulation involves convolving each heatmap with a 2D Gaussian kernel with the same standard deviation as generating the ground truth (i.e. 1.14µm), which helps to smooth the output.

Blob detection is performed using OpenCV, which employs an algorithm that thresholds the heatmap at multiple levels, and extracts connected components based on their properties, such as size and shape. To associate a ‘confidence score’ to each detection, we look up the value in the Gaussian modulated output heatmap at that coordinate.


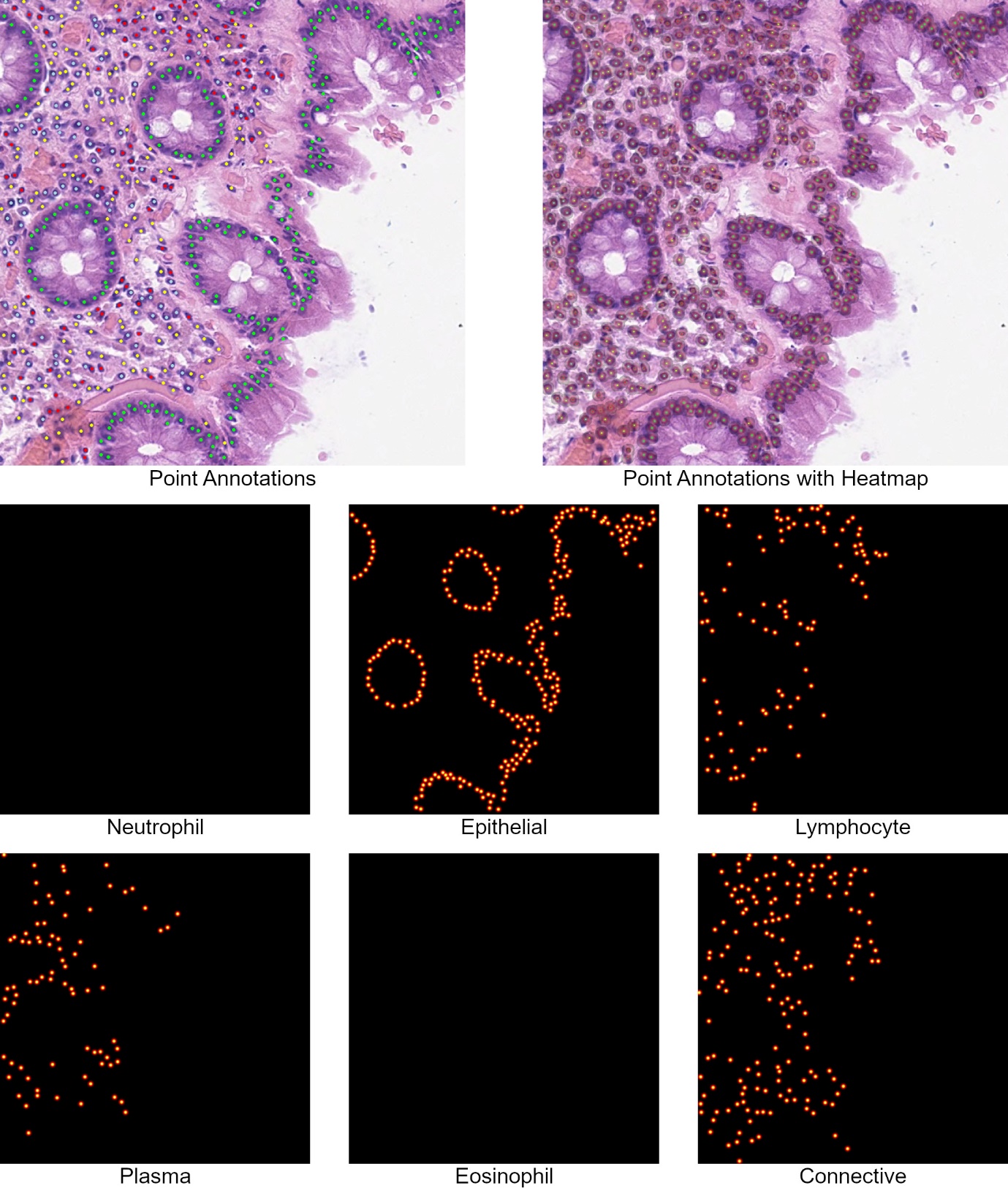


Figure S4: Illustration of the generated ground truth heatmaps used to perform point detection with a semantic segmentation network. Example from the Lizard^44^ dataset.

**Local Field Size Computation**

The side length of the square local regions, *l*, was selected such that the area matched that of the circular HPFs. Given the radius of the HPF is *r* = 0.275mm, we solved for *l* as follows:

$$l^{2}=\pi r^{2}$$

$$l=\sqrt{\pi\cdot{0.275}^{2}}$$

$$\approx0.4874mm^{2}$$

Figure S5 illustrates the effective amount of tissue contained within a HPF and one of our square fields. The total amount of tissue contained within each image is approximately equal.


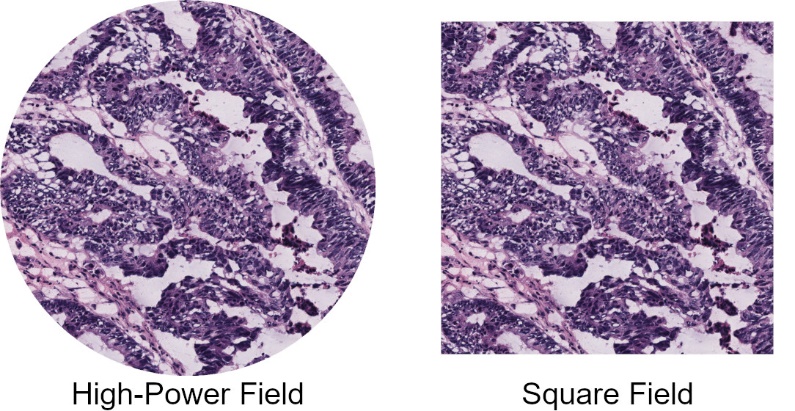


Figure S5: Illustration of local field sizes analysed manually and by our algorithm. The manually analysed high-power fields have a diameter of 0.55mm, whilst our algorithm analyses square fields with a side length of 0.4874mm^2^. These fields both contain approximately the same tissue area.

**TIL Method Flagging Criteria**

To reduce the chance of unreliable TIL scores produced by our system, we identified a set of criteria outlining situations that flag the produced score for recommended review by an expert. This was primarily implemented to identify abnormal situations that would typically require further analysis in pathology practice. Specifically, a WSI was flagged for review if any of the following were true:

- Not enough area to reliably analyse: If too little broad tumour (<20mm^2^) or no local areas of tumour were detected, then there may not be enough tumour analysed to make a representative iTIL score for the slide. In practice, this may require pathologists to look at other slides from the patient to complete their analysis.
- Excess necrosis detected: If an excess of necrosis was detected (>20% of TSN model predictions), this may suggest that the slide has a higher than expected amount of necrosis, and that analysing a second slide may be beneficial to fully capture the tumour.
- Excess iTILs detected: If more iTILs were detected than typically expected (globally >1000 iTILs per-mm^2^ tumour), this may suggest that tangentially sliced tumour nuclei are present. In practice, these areas may not be scored due to their appearance, and other slides may also be needed to make a complete assessment.

All criteria and thresholds were developed alongside discussions with pathologist DW by manually reviewing slides and model predictions.

Finally, we flag cases from patients where we could not find 5 local tiles from all of their available WSIs (or in cases where all of their WSIs were flagged). This decision was motivated by the approach taken by Williams et al.^6^, who selected at minimum 5 HPFs when analysing a case.

To ensure that the removal of flagged cases from survival analysis was not the cause of good stratification, we performed a survival analysis on the Austin-CRC and RNSH-CRC cohorts with the inclusion of all flagged cases. Cases that had no local fields to analyse were set to be low risk, with the assumption that there was minimal/no tumour on the slide.

Results from the survival analysis in each cohort can be seen in Figure S6. In terms of hazard ratio, including the flagged cases enhanced the survival model from HR 2.05, 95% CI 1.46–2.88; p<0.0001 to HR 2.10, 95% CI 1.52–2.91; p<0.0001 in the Austin-CRC cohort (5-Year RFS), and HR 1.86, 95\%CI 1.45–2.39; p<0.0001 to HR 1.96, 95% CI 1.53–2.50; p<0.0001 in the RNSH-CRC cohort (5-Year OS). This result suggests that the TIL scores generated on flagged cases are still reliable, and further shows that the flagging system acts to identify abnormal situations.


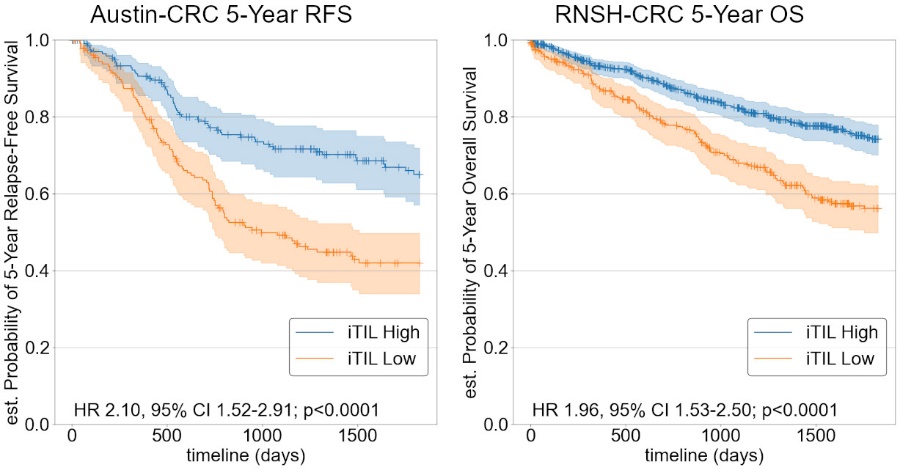


Figure S6: Kaplan-Meier curves illustrating 5-Year RFS on the Austin-CRC cohort and 5-Year OS on the RNSH-CRC cohort stratified by the AI iTIL score including all flagged cases.

**TIL Detection Confidence Threshold Selection**

An important decision when using an object detection model in practice is what (if any) confidence score threshold should be applied to refine the set of detections. In general, it is expected the model assigns a higher confidence score to detections that are more likely to be correct. To minimise false positive detections, confidence score filtering is commonly applied to reject detections with low confidence. One approach to select this threshold in a data-driven way is to find the confidence score threshold that maximises the F1 score (the harmonic mean of precision and recall), thus balancing the minimisation of both false positives and false negatives.

The best results achieved when optimising the F1 score are summarised in Table S3. Applying a confidence score threshold of 0.3335 resulted in an F1 score of 0.6014, which we rounded to 0.35. This rounding decision was made for two main reasons. Firstly, selecting such a precise value could risk overfitting to the model’s training data and might not be the ideal threshold for new data. Secondly, we aimed to reject more false positives (increasing precision at the cost of decreased recall) to have greater confidence that the detections classified as TILs were indeed TILs. This rounded value of 0.35 was applied in our TIL scoring method.

For visualisation purposes, we also generated a precision-recall (PR) curve to further illustrate this trade-off (Figure S7). Each point on the curve represents the precision and recall values achieved by applying a specific confidence score threshold. The background contours indicate the F1 score at each point, with higher F1 scores (and thus higher precision and recall) achieved as the curve approaches the top-right corner. As seen in the figure, as the confidence threshold increases (closer to 1), recall decreases (since fewer positive examples are retained), but precision increases (as the remaining examples are more likely to be true positives).

| **Confidence Threshold** | **F1** | **Precision** | **Recall** |
| --- | --- | --- | --- |
| 0.3335 | 0.6014 | 0.5492 | 0.6645 |
| 0.35 | 0.5982 | 0.5583 | 0.6444 |

Table S3: Metrics corresponding to the F1 score achieved by applying a confidence score threshold to TIL detections from the test set regions in the Austin-CRC + CPTAC-COAD datasets. The confidence threshold of 0.3335 maximises the F1 score, whilst the threshold of 0.35 achieves a similarly high F1 score by increasing precision at the cost of recall.


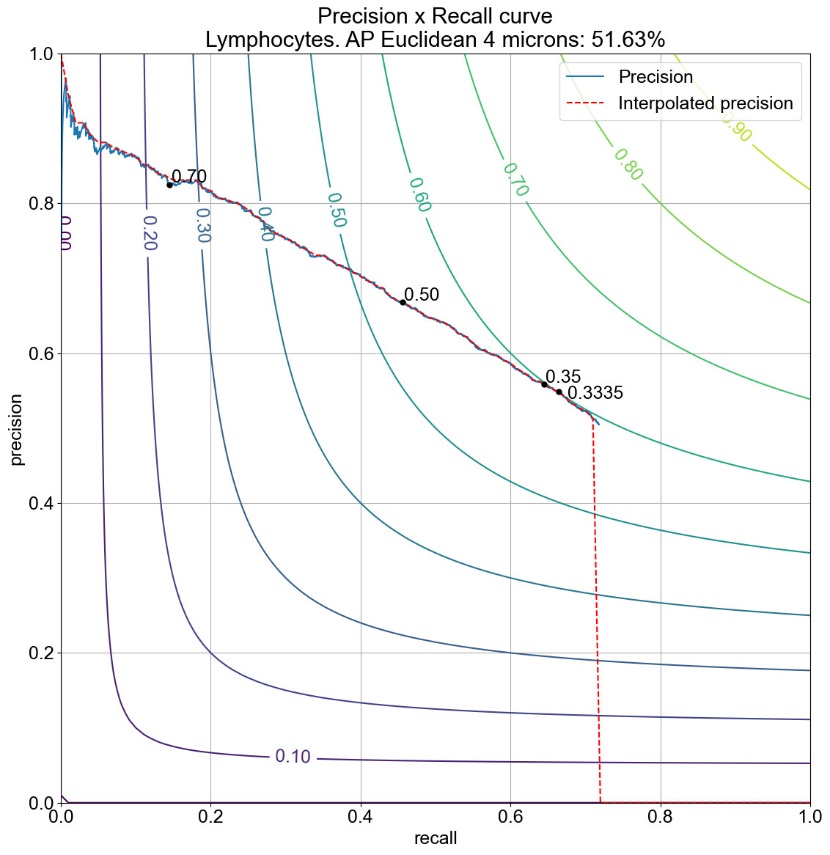


Figure S7: The PR curve corresponding to the trained TIL detection model. Contours represent the F1 score at that point on the plot. Points illustrate the values of precision and recall at 0.3335, 0.35, 0.5, and 0.7 confidence score thresholds.

***Deep Learning Segmentation Model Evaluation Results***

We report the F1 score, along with precision, recall, and Intersection over Union (IoU), to assess the performance of our segmentation models. IoU quantifies the overlap between predicted and ground truth segmentation masks. For a given class, it is computed by dividing the number of correctly predicted pixels (where both the predicted and ground truth masks agree on the classification) by the total number of pixels in either the predicted or the ground truth mask for that class. This is expressed as:

$$IoU=\frac{\left| A\cap B \right|}{\left| A\cup B \right|}$$

where *A* represents the predicted mask, and *B* represents the ground truth mask.

|  | **F1** | **Precision** | **Recall** | **IoU** |
| --- | --- | --- | --- | --- |
| **Tumour** | 0.9220 | 0.9609 | 0.8862 | 0.8624 |
| **Normal** | 0.9781 | 0.9650 | 0.9915 | 0.9577 |
| **Average** | 0.9500 | 0.9630 | 0.9388 | 0.9100 |

Table S4: Per-class and average metrics when evaluating the broad tumour segmentation model on the combined Austin-CRC + PAIP2020 test set.

|  | **F1** | **Precision** | **Recall** | **IoU** |
| --- | --- | --- | --- | --- |
| **Tumour** | 0.8812 | 0.9266 | 0.8400 | 0.7881 |
| **Stroma** | 0.9101 | 0.8647 | 0.9604 | 0.8350 |
| **Necrosis** | 0.6407 | 0.8729 | 0.5061 | 0.4714 |
| **Average** | 0.8107 | 0.8881 | 0.7688 | 0.6981 |

Table S5: Per-class and average metrics when evaluating the TSN segmentation model on the combined Austin-CRC + CPTAC-COAD test set.

**Discovery Cohort KM Curves**


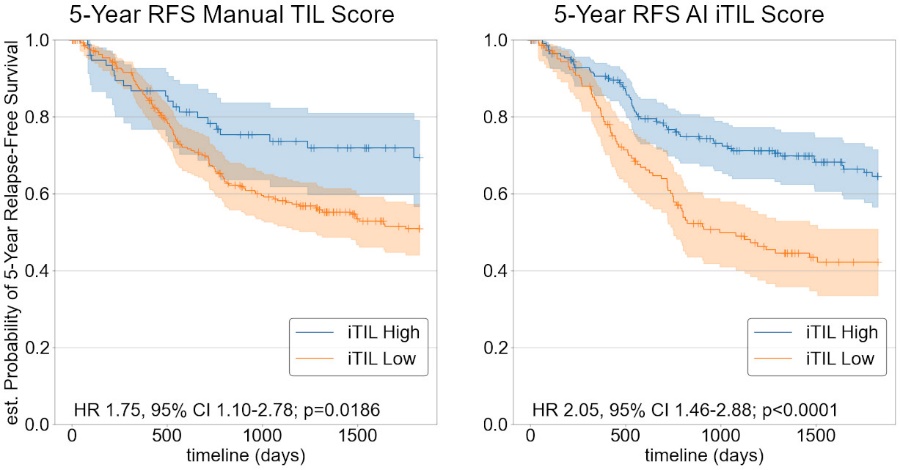


Figure S8: Kaplan-Meier curves illustrating 5-Year RFS on the Austin-CRC cohort stratified by the manual assessment made by pathologist DW and AI iTIL score. The AI-derived metric better stratifies the entire cohort by relapse risk.

**MCO-CRC Multivariate Analysis**

| **MCO-CRC** | **Univariate** | | | | **Multivariate** | | | |
| --- | --- | --- | --- | --- | --- | --- | --- | --- |
| **5-Year RFS** | **n** | **HR** | **95% CI** | **p** | **n** | **HR** | **95% CI** | **p** |
| Age (Decades) | 885 | 0.94 | 0.84-1.06 | 0.349 | 840 | 0.88 | 0.76-1.02 | 0.083 |
| Gender (Female vs Male) | 885 | 0.71 | 0.52-0.97 | 0.030* | 840 | 0.74 | 0.54-1.02 | 0.064 |
| Site (Proximal vs Distal) | 882 | 0.63 | 0.46-0.88 | 0.007* | 840 | 1.02 | 0.70-1.50 | 0.900 |
| Stage (III vs II) | 885 | 4.38 | 3.07-6.25 | <0.001* | 840 | 4.66 | 2.94-7.39 | <0.001* |
| T Stage (4 vs 1-3) | 885 | 2.61 | 1.91-3.56 | <0.001* | 840 | 2.16 | 1.54-3.02 | <0.001* |
| N Stage (2 vs 0-1) | 885 | 3.43 | 2.49-4.74 | <0.001* | 840 | 1.49 | 1.02-2.17 | 0.038* |
| Nodes Examined (<12 vs 12+) | 885 | 1.42 | 1.01-1.99 | 0.042* | 840 | 1.68 | 1.17-2.41 | 0.005* |
| Adjuvant Chemotherapy (yes vs no) | 867 | 1.47 | 1.08-1.99 | 0.014* | 840 | 0.41 | 0.28-0.60 | <0.001* |
| Grade (high vs low) | 885 | 1.54 | 1.11-2.14 | 0.010* | 840 | 1.30 | 0.92-1.86 | 0.141 |
| Lymphovascular Invasion (yes vs no) | 877 | 2.72 | 2.00-3.71 | <0.001* | 840 | 1.52 | 0.96-2.40 | 0.075 |
| Extramural Venous Invasion (yes vs no) | 859 | 2.54 | 1.86-3.47 | <0.001* | 840 | 1.13 | 0.72-1.77 | 0.605 |
| MMR Status (pMMR vs dMMR) | 885 | 2.48 | 1.43-4.28 | 0.001* | 840 | 2.25 | 1.19-4.28 | 0.013* |
| AI iTIL Risk (high vs low) | 885 | 1.60 | 1.17-2.21 | 0.004* | 840 | 1.15 | 0.82-1.62 | 0.405 |
| **MCO-CRC** |  |  |  |  |  |  |  |  |
| **5-Year OS** | **n** | **HR** | **95% CI** | **p** | **n** | **HR** | **95% CI** | **p** |
| Age (Decades) | 885 | 1.43 | 1.27-1.61 | <0.001* | 840 | 1.35 | 1.18-1.55 | <0.001* |
| Gender (Female vs Male) | 885 | 0.67 | 0.52-0.86 | 0.002* | 840 | 0.63 | 0.48-0.83 | <0.001* |
| Site (Proximal vs Distal) | 882 | 0.80 | 0.61-1.04 | 0.096 | 840 | 0.81 | 0.59-1.11 | 0.192 |
| Stage (III vs II) | 885 | 1.97 | 1.53-2.55 | <0.001* | 840 | 2.29 | 1.64-3.22 | <0.001* |
| T Stage (4 vs 1-3) | 885 | 2.15 | 1.66-2.80 | <0.001* | 840 | 1.98 | 1.49-2.63 | <0.001* |
| N Stage (2 vs 0-1) | 885 | 2.06 | 1.54-2.76 | <0.001* | 840 | 1.44 | 1.02-2.04 | 0.040* |
| Nodes Examined (<12 vs 12+) | 885 | 1.88 | 1.44-2.45 | <0.001* | 840 | 1.77 | 1.34-2.36 | <0.001* |
| Adjuvant Chemotherapy (yes vs no) | 867 | 0.69 | 0.53-0.90 | 0.006* | 840 | 0.41 | 0.29-0.58 | <0.001* |
| Grade (high vs low) | 885 | 1.61 | 1.23-2.10 | <0.001* | 840 | 1.37 | 1.01-1.85 | 0.040* |
| Lymphovascular Invasion (yes vs no) | 877 | 1.85 | 1.44-2.38 | <0.001* | 840 | 1.46 | 1.00-2.14 | 0.053 |
| Extramural Venous Invasion (yes vs no) | 859 | 1.79 | 1.37-2.34 | <0.001* | 840 | 1.03 | 0.69-1.52 | 0.900 |
| MMR Status (pMMR vs dMMR) | 885 | 1.13 | 0.80-1.59 | 0.480 | 840 | 1.10 | 0.72-1.67 | 0.654 |
| AI iTIL Risk (high vs low) | 885 | 1.48 | 1.13-1.93 | 0.004* | 840 | 1.31 | 0.98-1.75 | 0.066 |

Table S6: Univariate and multivariate results for MCO-CRC cohort 5-Year RFS and 5-Year OS. *p<0.05.

**Stage II Clinical High Risk Breakdown**

|  | **RNSH-CRC** | **MCO-CRC** |
| --- | --- | --- |
|  | **n (%)** | **n (%)** |
| Cases | 533 (49.8%) | 464 (52.4%) |
| Clinical High Risk | 290 (54.4%) | 267 (57.5%) |
| pMMR | 192 (66.2%) | 197 (73.8%) |
| Clinical Low Risk | 243 (45.6%) | 197 (42.5%) |
| pMMR | 181 (74.5%) | 162 (82.2%) |

Table S7: Breakdown of Stage II cases with clinical high and low risk information available in the RNSH-CRC and MCO-CRC cohorts. This includes counts of pMMR cases per-group.

**Stage II Clinical High Risk Multivariate Analysis**

|  | **Univariate** | | | | **Multivariate** | | | |
| --- | --- | --- | --- | --- | --- | --- | --- | --- |
|  | **n** | **HR** | **95% CI** | **p** | **n** | **HR** | **95% CI** | **p** |
| Age (Decades) | 557 | 1.69 | 1.38-2.08 | <0.001* | 554 | 1.84 | 1.48-2.30 | <0.001* |
| Gender (Female vs Male) | 557 | 0.71 | 0.49-1.02 | 0.060 | 554 | 0.76 | 0.52-1.12 | 0.162 |
| Site (Proximal vs Distal) | 554 | 0.55 | 0.38-0.79 | 0.001* | 554 | 0.52 | 0.34-0.81 | 0.003* |
| T Stage (4 vs 1-3) | 557 | 1.43 | 0.99-2.08 | 0.060 | 554 | 2.00 | 1.34-2.98 | <0.001* |
| Nodes Examined (<12 vs 12+) | 557 | 2.10 | 1.45-3.04 | <0.001* | 554 | 2.13 | 1.41-3.22 | <0.001* |
| Grade (high vs low) | 557 | 0.99 | 0.68-1.45 | 0.971 | 554 | 1.79 | 1.16-2.75 | 0.008* |
| Lymphovascular Invasion (yes vs no) | 557 | 1.01 | 0.69-1.48 | 0.947 | 554 | 1.12 | 0.70-1.78 | 0.647 |
| Extramural Venous Invasion (yes vs no) | 557 | 1.08 | 0.72-1.62 | 0.724 | 554 | 1.33 | 0.79-2.23 | 0.281 |
| MMR Status (pMMR vs dMMR) | 557 | 1.32 | 0.87-2.02 | 0.194 | 554 | 0.96 | 0.57-1.61 | 0.864 |
| AI iTIL Risk (high vs low) | 557 | 2.21 | 1.53-3.20 | <0.001* | 554 | 2.00 | 1.34-2.96 | <0.001* |

Table S8: Univariate and multivariate results for Stage II Clinical High Risk combined RNSH-CRC + MCO-CRC cohort 5-Year OS. *p<0.05.

**Stage II pMMR Clinical High Risk Multivariate Analysis**

|  | **Univariate** | | | | **Multivariate** | | | |
| --- | --- | --- | --- | --- | --- | --- | --- | --- |
|  | **n** | **HR** | **95% CI** | **p** | **n** | **HR** | **95% CI** | **p** |
| Age (Decades) | 389 | 1.70 | 1.34-2.17 | <0.001* | 386 | 1.80 | 1.40-2.31 | <0.001* |
| Gender (Female vs Male) | 389 | 0.88 | 0.58-1.33 | 0.540 | 386 | 0.98 | 0.64-1.51 | 0.932 |
| Site (Proximal vs Distal) | 386 | 0.49 | 0.31-0.79 | 0.004* | 386 | 0.48 | 0.29-0.78 | 0.003* |
| T Stage (4 vs 1-3) | 389 | 1.47 | 0.96-2.25 | 0.076 | 386 | 2.33 | 1.46-3.73 | <0.001* |
| Nodes Examined (<12 vs 12+) | 389 | 2.23 | 1.45-3.43 | <0.001* | 386 | 2.42 | 1.49-3.92 | <0.001* |
| Grade (high vs low) | 389 | 1.09 | 0.67-1.78 | 0.728 | 386 | 1.76 | 1.05-2.93 | 0.031* |
| Lymphovascular Invasion (yes vs no) | 389 | 0.88 | 0.57-1.37 | 0.570 | 386 | 1.06 | 0.61-1.85 | 0.834 |
| Extramural Venous Invasion (yes vs no) | 389 | 0.90 | 0.57-1.43 | 0.653 | 386 | 1.28 | 0.70-2.34 | 0.427 |
| AI iTIL Risk (high vs low) | 389 | 2.38 | 1.57-3.61 | <0.001* | 386 | 2.17 | 1.42-3.33 | <0.001* |

Table S9: Univariate and multivariate results for Stage II pMMR Clinical High Risk combined RNSH-CRC + MCO-CRC cohort 5-Year OS. *p<0.05.

**Continuous iTIL Score Distribution**


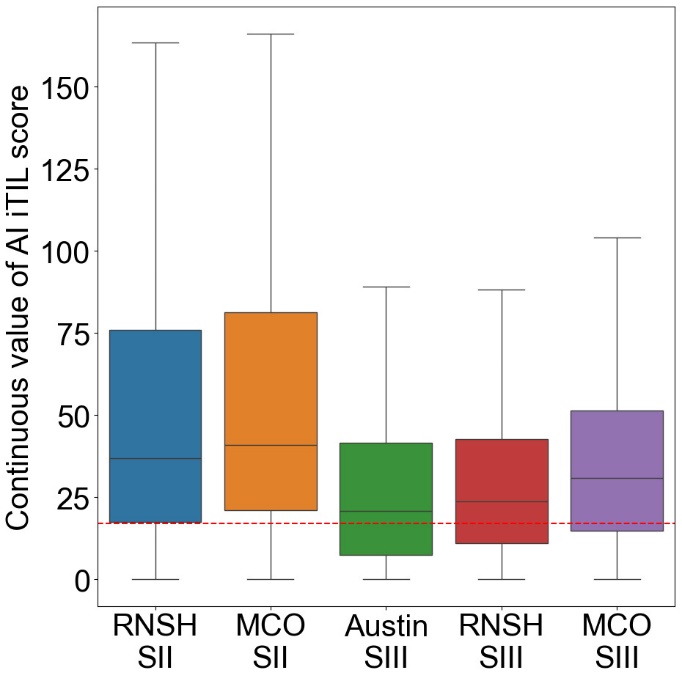


Figure S9: Box plot illustrating the distribution of the continuous AI iTIL metric value per-stage in each cohort (outliers not shown on plot). The horizontal dotted line represents the cut-off threshold used to stratify patients by risk. Stage II patients were found to generally have more iTILs than Stage III patients. Given Stage II patients have better outcomes than Stage III, this finding is consistent with higher iTIL counts resulting in better patient outcome. The median iTIL count in the MCO-CRC Stage III cohort was higher than Stage III counts from other cohorts, which may explain why significant stratification was not seen in MCO-CRC Stage III.

**Model Inference Examples**


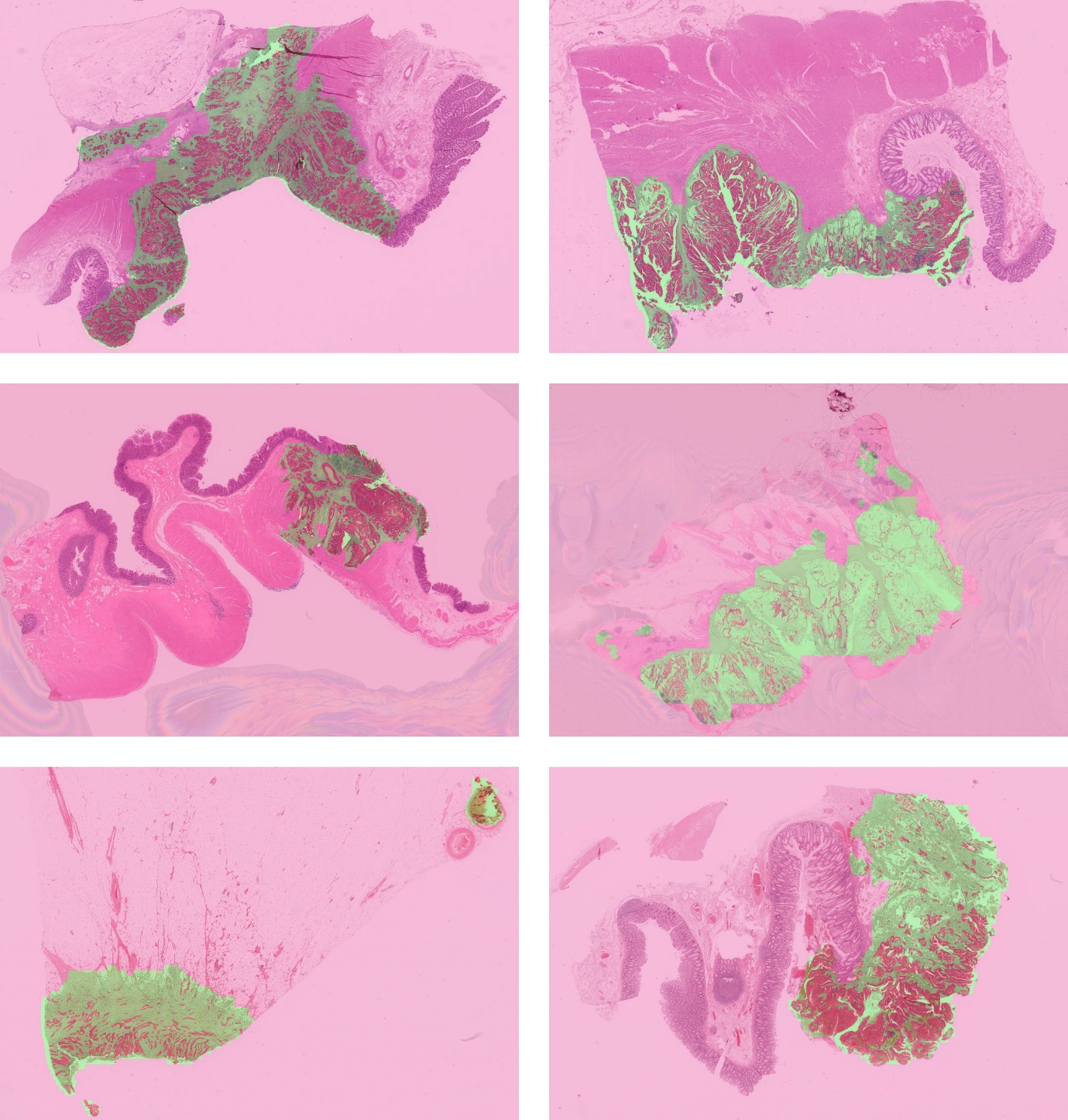


Figure S10: Illustrations of predictions made by the TIL method on data used in survival analysis with and without mucinous histology. Left column: Non-Mucinous, right column: Mucinous. Top row: Austin-CRC. Middle row: RNSH-CRC. Bottom row: MCO-CRC. Dark pink represents areas the broad tumour model did not detect as broad tumour foreground. Red, green, and black overlay represents predictions of tumour, stroma, and necrosis respectively from the TSN model.
